# Metabolic Mechanisms in Electroconvulsive Therapy for Schizophrenia: Role, Potential and Future Directions

**DOI:** 10.3390/ijms27041749

**Published:** 2026-02-11

**Authors:** Wenjing Ding, Tianhao Bao

**Affiliations:** Mental Health Center, Kunming Medical University, Kunming 650034, China; 20201453@kmmu.edu.cn

**Keywords:** metabolic disorders, schizophrenia, electroconvulsive therapy, mechanism, carbohydrates, lipids, amino acids, nucleotides

## Abstract

The metabolism of the four major substances—glucose, lipids, amino acids, and nucleotides—constitutes the most prominent metabolic phenotype of schizophrenia. The pathological axis shared by these substances involves energy pathway imbalances, redox stress, immune-inflammatory activation, and abnormalities in neurotransmitter synthesis/degradation. Existing research confirms that key metabolites within these pathways hold potential as biomarkers for diagnosis or progression monitoring. In recent years, electroconvulsive therapy (ECT) has been shown to improve psychotic symptoms while exerting broad regulatory effects on neurogenesis, immune homeostasis, and the hypothalamic–pituitary–target gland axis, though its precise mechanisms remain unclear. Recent studies indicate that ECT treatment can also regulate changes in brain and peripheral metabolism. We propose an integrated “metabolism-immunity-neuroendocrine” hypothesis to systematically elucidate how metabolic reprogramming during ECT treatment cascades sequentially to the immune, neural, and endocrine systems, thereby revealing the molecular basis of its antipsychotic effects. Furthermore, we conduct a comparative analysis of the effects of antipsychotic drugs on the same metabolic network and explore the universality and specificity of metabolic regulation in other physical therapies (such as rTMS, tDCS) and psychiatric disorders like depression and bipolar disorder. This research aims to provide novel biomarkers and intervention targets for the precision diagnosis and treatment of schizophrenia.

## 1. Introduction

Schizophrenia (SCZ) is a complicated, long-term mental illness that has a significant impact on patients, families, and society as a whole due to its high incidence, recurrence, and disability rates [1]. In recent years, with the widespread adoption of multi-omics technologies, the role of metabolic mechanisms in the pathophysiology of schizophrenia has garnered increasing attention. The structural and functional alterations in the schizophrenic brain may be attributed to metabolic abnormalities. Early brain development metabolic mechanisms have an impact on the structure and connections of the brain, and they overlap with hereditary factors in the pathophysiology of schizophrenia [2,3], affecting brain structure and connectivity between regions. Schizophrenia patients exhibit reduced functional activity in the medial prefrontal cortex (mPFC) alongside increased microglial activity [4,5], accompanied by impaired white matter structural integrity and reduced gray matter volume [6]. Furthermore, metabolic abnormalities change as the disease progresses. Altered lactate levels in schizophrenia patients reflect a shift in energy metabolism from aerobic oxidation to anaerobic glycolysis, which could serve as a biomarker for schizophrenia diagnosis [7]. Additionally, oxidative stress, inflammatory reactions, and neurotransmitter abnormalities are linked to metabolic imbalances; several metabolites have emerged as new treatment targets for schizophrenia. However, widely used antipsychotic medications frequently cause drug-induced metabolic syndrome [8] and worsen metabolic problems by inhibiting D2 receptors. A widespread physical treatment in psychiatric practice, electroconvulsive therapy (ECT) addresses the limits of drug-resistant schizophrenia while providing safety and effectiveness. In a recent study, ECT reduced IL-18 mRNA levels in schizophrenia patients, while kynurenine (KYN)/tryptophan (TRP) and kynurenic acid (KYNA)/KYN were significantly reduced in the low-inflammation group. These alterations correlated with improvements in negative symptoms [9], implying that ECT also affects brain and peripheral metabolic processes. However, the causal association between metabolic changes and ECT efficacy, as well as specific regulatory nodes and drug interactions, is still controversial. Further synthesis and exploration are needed to guide treatment optimization and intervention strategies.

In this paper, we first summarize the primary metabolic abnormalities in schizophrenia, categorized into glucose, lipid, amino acid, and nucleotide metabolism, elucidating the relationship between metabolism and the disease’s pathogenesis and symptoms. We then review how ECT treatment has historically exerted its antipsychotic effects on the neurological, immune, and endocrine systems in schizophrenia. Building on this foundation, we discuss how metabolic reprogramming during ECT treatment influences these systems. We then compare the effects of antipsychotic drugs on metabolic networks and review the role of metabolic regulation in other physical therapies and psychiatric disorders. Finally, we summarize and identify potential future research and practice trends and pathways that may prove significant.

## 2. The Role of Metabolic Mechanisms in the Pathophysiological Processes of Schizophrenia

### 2.1. Schizophrenia and Abnormal Glucose Metabolism

Glycolysis in the cytoplasm breaks down glucose into pyruvate or lactate, which is subsequently oxidatively phosphorylated in the mitochondria. This pathway generates the high-energy molecule adenosine triphosphate (ATP), which drives brain function. Additionally, glucose participates in antioxidant stress through the pentose phosphate pathway (PPP), provides substrates for neuronal glycolipid and glycoprotein synthesis, and generates glutamate and subsequent key neurotransmitters such as gamma-aminobutyric acid (GABA) via the tricarboxylic acid (TCA) cycle [10,11]. As a result, abnormalities in any stage of glucose metabolism can have a significant impact on brain development and function.

Numerous clinical studies consistently demonstrate that altered glucose metabolism exists in schizophrenia patients throughout the early stages of the disease [12,13,14], presenting as higher fasting blood glucose, insulin resistance, and impaired glucose tolerance [15,16,17,18]. This is unrelated to antipsychotic-induced metabolic syndrome [13,19], but it is strongly linked to aberrant brain tissue energy metabolism caused by mitochondrial dysfunction and redox imbalance [20,21,22]. Some genetic studies provide strong evidence for the above claims. Whole-genome studies have clearly demonstrated that schizophrenia risk genes contribute to pathogenesis by regulating glucose metabolism, with the most significantly enriched pathways directly linked to glucose homeostasis and insulin secretion [2]. Mendelian randomization analysis revealed that genetic variants that raise fasting insulin levels significantly increase disease risk with established causal directionality [23], and animal model mice with mutations in the candidate gene *Tmem108* also exhibit symptoms of impaired glucose tolerance and insulin resistance [24]. GLUT1 and GLUT3, members of the glucose transporter (GLUT) family, play a crucial role in neuronal glucose uptake [25]. In schizophrenia, cerebral insulin resistance inhibits GLUT1/3-mediated glucose uptake, but systemic hypoglycemia upregulates GLUT1/3 expression, indicating decoupling between peripheral hyperglycemia and impaired cerebral glucose utilization. A postmortem study revealed significant downregulation of GLUT1/3 mRNA in the dorsolateral prefrontal cortex (DLPFC) of schizophrenia patients (*n* = 16) [26], corroborating this observation. Several studies have further indicated that this exacerbates negative symptoms and promotes symptom transformation into chronic damage [27,28]. Furthermore, mTOR pathway dysfunction—a pathogenic factor in schizophrenia [29]—disrupts GLUT1 receptor translocation, exacerbating neuronal insulin resistance and ATP deficiency [30].

Extensive clinical evidence indicates that abnormalities in glucose metabolism enzymes are prevalent in the brains of schizophrenic patients. Previous studies scanning the schizophrenia dataset from the National Institute of Mental Health (NIMH) revealed that schizophrenia susceptibility genes are associated with enzymes involved in aerobic glycolysis, such as 6-phosphofructokinase-2/fructose-2,6-bisphosphatase 2 (PFKFB2), hexokinase 3 (HK3), and pyruvate kinase 3 (PK3) [31]. Postmortem studies showed lower levels of aldehyde dehydrogenase C (ALDOC) and α-enolase (ENO1) in patients’ hippocampus [32], as well as considerably lower mRNA expression of hexokinase 1 (HK1) and phosphofructokinase (PFK1) in DLPFC pyramidal neurons, while lactate/pyruvate transporter (MCT1) increased [26]. Pyruvate is converted to acetyl-CoA via pyruvate dehydrogenase (PDH), and one study showed lower PDH β-subunit levels in the striatum of schizophrenia patients compared to healthy controls [33]. In addition, one study revealed a negative correlation between mitochondrial HK1 activity and glucose-6-phosphate dehydrogenase (G6PD) activity in the parietal sensory cortex (BA7) in schizophrenia patients [34], suggesting that abnormal glucose metabolism may coexist with mitochondrial damage induced by oxidative stress. Disrupted glucose metabolism enzyme profiles in the peripheral blood mononuclear cells [12] and gut microbiota [35] of schizophrenia patients are consistent with the previous findings. Additionally, decreased HK activity in PFC, increased malate dehydrogenase (MDH) activity, and decreased lactate dehydrogenase (LDH) activity in the striatum were similarly observed in schizophrenia model rats [36], which collectively contribute to impaired glucose utilization, lactate accumulation, and white matter lesions [37]. Although most of the above studies lack reproducibility, they collectively suggest that glucose metabolism in schizophrenia may exhibit an imbalance characterized by “enhanced glycolysis and inhibited tricarboxylic acid cycle.”

The majority of ATP produced by glucose metabolism is used by brain neurons to maintain synaptic excitement. Cognitive performance may be hampered by impaired glucose utilization and lactic acid accumulation. The astrocyte-neuron lactate shuttle (ANLS) is the brain’s crucial mechanism for providing cognitive energy [38,39,40]. Monocarboxylate transporters (MCTs) carry lactate, which is produced by astrocytes through glycolysis, to neurons. Lactate dehydrogenase (LDH) transforms lactate into pyruvate, which is then processed in mitochondria via the tricarboxylic acid (TCA) cycle and oxidative phosphorylation (OXPHOS) to generate ATP [41]. Research has confirmed abnormal bioenergetic coupling between astrocytes and neurons in schizophrenia [42], and abnormal lactate metabolism is a necessary component of ANLS imbalance [43,44]. This elevated brain lactate may be associated with a shift in energy metabolism from the TCA cycle and OXPHOS toward greater reliance on glycolysis, similar to the Warburg effect reported in cancer cells [45,46]. Extensive research confirms that lactate levels in schizophrenia patients are commonly elevated in multiple brain regions (DLPFC, striatum, hippocampus, etc.) [14,33,47,48,49], peripheral blood [50], and cerebrospinal fluid (CSF) [51,52,53,54,55], and are negatively related to a decreased pH [14,33,56,57]. Abnormal brain energy metabolism is often accompanied by elevated brain lactate levels; this phenomenon persists across schizophrenia-associated genetic models (DISC1 [58], 22q11.2 deletion [59]), cellular models (iPSCs) [49], and pharmacological models [56,60]. Consequently, lactate abnormalities reflect disturbances in energy metabolism. Lactic acid abnormalities in schizophrenia are independent of drug administration and correlate with early OXPHOS enzyme depletion [61] (PDH [33,62], α-ketoglutarate and citrate [53]), mitochondrial dysfunction, oxidative stress, and tissue hypoxia [63]. Recent evidence indicates that elevated lactate levels correlate with symptom severity [64]. Lactate reduction improves cognitive symptoms [65,66], making brain lactate a viable biomarker for early diagnosis and treatment monitoring of this disorder [7,67].

Glycosylation refers to the enzymatic binding of sugars to proteins and lipids. A missense mutation (A391T) in the schizophrenia-associated gene *SLC39A8* impairs serum manganese sensitivity, resulting in congenital n-glycosylation abnormalities in plasma [68,69]. The *ADAMTS9* and *PIGQ* genes are also linked to glycosylation abnormalities in schizophrenia [70]. Several studies have found abnormal glycosylating enzyme profiles in the brains of schizophrenia patients [71,72,73]. Additional studies indicate significant downregulation of synaptic plasticity glycoproteins in the DLPFC, such as PSA-NCAM [74] and PNN [75], alongside abnormalities in glutamate receptor AMPA and NMDA receptor subunits [76,77], glutamate transporters EAAT1 and EAAT2 [78], and GABAA receptors [79,80]. This evidence indicates abnormal glycosylation in schizophrenia. Advanced glycation end-products (AGEs) are the principal cause of carbonyl stress and characterize refractory disease states [81], including methylglyoxal (MG) and pentosyl in the brain. Endogenous secretory RAGE (esRAGE) protects cells from AGE toxicity [82]. One study demonstrated AGE accumulation and diminished esRAGE/sRAGE protective effects in schizophrenia patients [83]. Pyrovalerone, betaine, and glyoxalase 1 (GLO1) counteract carbonyl stress. Clinical evidence suggests elevated peripheral pentosidine levels, reduced pyridoxal [84,85] and betaine [86] levels, and decreased GLO1 activity in schizophrenia, suggesting a potential association with anxiety, depression-like behaviors, and symptom severity [87,88]. Although abnormal glycosylation and carbonyl stress are present in schizophrenia, studies investigating their relationship with disease phenotypes are scarce and require further validation.

Based on the above evidence, schizophrenia can be regarded as a disorder involving impaired energy metabolism. Under the combined influence of genetic susceptibility and environmental stressors, peripheral hyperglycemia coexists with central brain glucose metabolism abnormalities in schizophrenia. Abnormalities are observed in the glucose metabolism enzyme profile and lactate metabolism (Table 1), suggesting an energy metabolism imbalance shifting from the tricarboxylic acid cycle/oxidative phosphorylation to glycolysis. This interacts with common pathways involving oxidative stress, mitochondrial dysfunction, and carbonyl stress, ultimately impairing neuronal synaptic plasticity and neurotransmitter balance. These alterations are closely associated with psychotic symptoms and cognitive impairments, providing novel targets for early disease identification and metabolic intervention.

### 2.2. Schizophrenia and Abnormal Lipid Metabolism

Both the schizophrenic brain (neuronal membranes, myelin sheaths) and peripheral tissues (serum, liver, adipose tissue, etc.) have significant lipid metabolism problems. Multiple studies have shown that lipid abnormalities are a significant feature of the disease [89,90], and they are closely related to inflammation, oxidative stress, and energy metabolism imbalance. These abnormalities affect symptom severity, cognitive impairment, and prognosis [91].

Fatty acid and cholesterol generation in oligodendrocyte myelin inside the central nervous system are regulated by the schizophrenia susceptibility genes *SREBF1* and *SREBF2* [3,92]. The schizophrenia susceptibility genes *APOEε2* [93] and *G72/G30* [94] are both implicated in lipid abnormalities. According to the membrane lipid hypothesis of schizophrenia [95], inadequate phospholipid production or excessive breakdown is a pathogenic process that causes diminished membrane fluidity, poor synaptic plasticity, and neurotransmitter receptor dysfunction [96]. Phospholipids include phosphatidylserine (PS), phosphatidylethanolamine (PE), phosphatidylcholine (PC), lysophosphatidylethanolamine (LPE), lysophosphatidylcholine (LPC), and ethanolamine acylglycerol precursors. PC and sphingosine are primary synthesis ingredients for neurons and oligodendrocytes. Phospholipase A2 (PLA_2_) converts PE to LPE [97]. Lipid peroxidation increases PLA_2_ activity, triggering excessive degradation of membrane phospholipids and releasing pro-inflammatory mediators (e.g., arachidonic acid) [98]. Myelin sheaths are mostly composed of sphingolipids, which also include sulfatides and ceramides. Ceramides exert apoptotic and inflammatory effects, and phosphatidylserine enhances ceramide-induced cell death [73]. Extensive study has revealed widespread abnormalities in neuronal membrane phospholipids and myelin lipids in the brains of schizophrenic patients. A non-targeted lipidomics study revealed widespread decreases in PC, PE, and cardiolipin throughout frontal cortex gray matter, particularly in elderly patients [99], suggesting synthetic insufficiency. Multiple cohort studies consistently demonstrate elevated levels of sulfatides, N-acylphosphatidylserine, and phospholipid metabolites in the frontal cortex of schizophrenia patients [100,101,102]; this confirms abnormalities in membrane phospholipids and sphingolipids. Among these, only one study reported increased levels of choline acetaldehyde decarboxylase precursor and ethanolamine [100,101]. Ceramides decrease in gray matter [102] but increase in white matter [100], potentially due to differences in brain regions and lipid subclasses. Another study found significantly elevated concentrations of ceramides in both the white matter and gray matter of the prefrontal cortex in schizophrenia patients [103], reflecting heightened inflammatory and apoptotic signaling. Additionally, research has found abnormal concentrations of PC and PE metabolites in subcortical and cortical regions of schizophrenia patients, with cortical PC levels correlating with psychotic symptoms [104]. Collectively, phospholipid–sphingolipid network dysregulation disrupts neuronal signaling, myelin formation, and oligodendrocyte function, jointly driving the development of cognitive and psychotic symptoms in schizophrenia [90,105].

Fatty acids and their derivatives serve as core substrates for membrane structure and signaling molecules. Polyunsaturated fatty acids (PUFAs), which are rich in double bonds, are vulnerable to free radical attack. Lipid peroxidation can disrupt membrane permeability and damage mitochondria. Neuroactive steroids (cholesterol esters) are also considered potential therapeutic targets for psychiatric disorders [106]. Schizophrenic patients exhibit significantly elevated levels of free fatty acids, ceramides, and triglycerides in the frontal cortex [103,107], suggesting concurrent membrane lipid remodeling and oxidative stress. Some researchers propose that cerebral lipid abnormalities correlate with energy metabolism imbalance. Insufficient glucose supply causes the body to mobilize peripheral fat, resulting in compensatory increases in serum free fatty acids (FFAs) and the ketone body β-hydroxybutyrate (β-HB). Lipid peroxidation damages membrane lipids, and excess FFAs entering the brain exacerbate peroxidation through the release of free polyunsaturated fatty acids (PUFAs), which worsen oxidative damage and impair glucose utilization. This creates a vicious cycle of “energy deficit—lipolysis—re-damage.” Multiple studies confirm elevated levels of various fatty acids and ketone bodies in the serum/urine of schizophrenia patients [108], with β-HB positively correlated with fasting blood glucose and triglycerides [109], suggesting insufficient glucose supply and sustained hyperactivity in fatty acid catabolism. However, as the disease progresses, β-HB levels decrease when the body loses compensatory capacity [51,110]. Brain metabolites can be measured using combined proton and phosphorus magnetic resonance spectroscopy (^1^H/^31^P-MRS). Extensive research has validated the aforementioned pathways from an energy metabolism perspective. Phosphomonoester (PME) serves as a precursor for phospholipid synthesis, while phosphodiester (PDE) is a degradation metabolite. In first-episode, untreated schizophrenia patients, anterior cingulate PDE levels increase synchronously with high-energy phosphate, while PME levels decrease [111,112], suggesting reduced phospholipid synthesis and localized hypermetabolism during the acute phase. With disease progression or chronic medication use, studies have found a significant decrease in the total adenosine triphosphate (ATP)/phosphocreatine (PCr) ratio in the basal ganglia of schizophrenia patients, along with an increase in the PME/PDE ratio, due to lipid peroxidation and reduced energy demand [113]. Additional studies reveal widespread reductions in lipid metabolites (PME and PDE) and energy metabolites (PCr and Pi) across the bilateral prefrontal cortex, hippocampus, caudate nucleus, thalamus, and anterior cerebellum in patients, with these alterations positively correlating with PANSS and BPRS scores [112,114,115], indicating direct linkage between impaired membrane lipid turnover and symptom severity. The glutamatergic system may cross-regulate this lipid-energy axis: one study found that elevated Glu in the left prefrontal cortex of schizophrenia patients correlates with increased PME (membrane repair), while right-sided Glu elevation correlates with increased PDE (membrane degradation), corresponding to negative symptoms and cognitive deficits [116]. The endocannabinoid system (ECS) comprises cannabinoid receptor 1 (CB1R) and 2 (CB2R), with endogenous ligands including endocannabinoids and 2-arachidonoylglycerol (2-AG) [117]. Some studies indicate that CB1R expression is downregulated in brain tissue, endogenous cannabinoid levels are elevated, and the fatty acid:phospholipid:cholesterol ester ratio in the olfactory epithelial cells of schizophrenia patients is imbalanced, with enhanced lipid peroxidation. This phenomenon is not observed in long-term cannabis users [118,119,120], suggesting that ECS dysregulation is disease-specific rather than drug-induced, and the astrocytic ECS system holds potential to link lipid metabolism with neuroinflammation—a hypothesis requiring extensive validation. In summary, the schizophrenia brain exhibits a self-amplifying pathological loop: “glucose deficiency → fatty acid/ketone body compensation → lipid peroxidation → membrane lipid remodeling and energy depletion.” This process may also be accompanied by glutamatergic hyperactivity and ECS imbalance, leading to cognitive impairment and psychotic symptoms.

Schizophrenia’s peripheral lipid profile (serum, plasma, platelets, and red blood cells) shows a stable phenotype with decreased membrane phospholipids, increased storage fats, and lipid peroxidation. This profile has a significant correlation with illness features, cognitive impairment, and treatment response [121], with inflammation, oxidative stress, and an imbalance in energy metabolism all playing crucial roles. Multiple studies using targeted or untargeted lipidomics platforms consistently demonstrate elevated levels of PC, PE, LPC, LPE, N-acylsphingomyelin, phospholipidylcholine plasma alcohols (plas-PCs), and phosphoethanolamine plasma alcohols (plas-PEs) [122,123,124] in various blood components of schizophrenia patients (including twins [125], first-episode untreated individuals, and relapse-off-medication cases [126,127]), regardless of age or gender. Notably, the reduction in LPC in the serum of monozygotic twins with schizophrenia positively correlates with cortical gray matter density and cognitive scores, suggesting persistent depletion of membrane phospholipids. Consistent with brain findings, peripheral tissues exhibit bidirectional upregulation of fats and sphingolipids. Triglycerides (TG) are elevated across studies, with saturated-chain TG further accumulating after antipsychotic treatment [128], while sphingomyelin (SM) results showed heterogeneity. However, the “low SM-high symptom” pattern was replicated in patients’ red blood cells post-treatment, potentially due to specific red blood cell membrane lipid clusters being associated with dopamine dysfunction [129]. Free fatty acids and cholesterol profiles are similarly disrupted, with elevated serum levels of 16 FFAs, MUFAs, and some PUFAs [89,130]. First-episode treatment-resistant patients exhibit increased serum TC, LDL, and TG [131]. Cohort studies also show that serum glycerophospholipids (GP), sphingomyelin (SP), and glycerolipids (GL) decrease, while ceramides, LPC, and TG monomers increase [90], suggesting active lipolysis–reesterification cycles. Concurrently, oxidized lipids significantly increase in red blood cells, while ether lipids and PUFAs decrease, directly confirming membrane peroxidation damage [132]. Furthermore, the gut–brain axis may contribute to peripheral lipid reprogramming. Fecal metagenomic analyses reveal an altered abundance of short-chain fatty acid (SCFA)-producing bacteria, enrichment of glycerophospholipid metabolic pathways, and reduced abundance of fatty acid synthesis rate-limiting enzyme acetyl-CoA carboxylase (ACC) genes [35,133]. Interestingly, first-episode schizophrenia patients can be distinguished from healthy controls by elevated LPC, reduced PC, and decreased SM levels, with PC lipid levels negatively correlated with disease severity [134]. CHR individuals exhibit low unsaturation TG↑ and ether phospholipids↓, predictive of psychotic conversion, with lower sphingomyelin in males [135]. Low SM/high PS clusters in red blood cells correlate negatively with PANSS cognitive factors. Lipid profiles in plasma samples from schizophrenia spectrum disorder patients (with comorbidities) interact with inflammation, though schizophrenia-specific findings remain undiscussed [136]. Collectively, the “membrane phospholipid depletion-lipid mobilization-peroxidative injury” pattern persists stably in peripheral tissues throughout the course of schizophrenia, exhibiting a close association with cognitive impairment. However, the precise interactions between this pattern and inflammation, oxidative stress, and energy metabolism imbalances require further investigation through large-scale studies.

In summary, schizophrenia susceptibility genes downregulate phospholipid/sphingolipid synthesis. This, combined with inflammation, oxidative stress, and impaired glucose metabolism, leads to a vicious cycle of “membrane lipid abnormalities (Table 2) and lipid peroxidation-energy supply insufficiency” occurring simultaneously in both central and peripheral tissues. Concurrently, abnormalities in myelin sheath and synaptic membrane structures ultimately manifest as cognitive deficits and psychotic symptoms. These abnormalities serve as a critical foundation for illness initiation and progression while also presenting novel biomarkers and therapeutic targets for clinical diagnosis and treatment.

### 2.3. Schizophrenia and Abnormal Amino Acid Metabolism

Amino acids are the fundamental building blocks of proteins and peptides. Among these, glutamic acid (Glu), glycine (Gly), serine (Ser), and tryptophan (Trp) are involved in immunological responses, redox homeostasis, and energy metabolism in addition to acting as neurotransmitters or their precursors. Clinical studies indicate that the pathophysiology of schizophrenia patients is correlated with aberrant serum levels of biogenic amines (BAs) and amino acids (AAs) [137].

The dopamine hypothesis of schizophrenia proposes that excessive striatal dopamine (DA) activity, along with insufficient frontal lobe DA, is responsible for psychotic symptoms [138,139,140]. Phenylalanine hydroxylase (PAH) converts phenylalanine to tyrosine, which is then produced into DA. In both first-episode and chronic schizophrenia patients, chronic inflammation and oxidative stress can impair PAH function, resulting in increased plasma phenylalanine levels and limited DA production [141]. Furthermore, the schizophrenia susceptibility gene *DISC1* further disrupts DA biosynthesis via the “serine phosphorylation–tyrosine hydroxylase” pathway, suggesting that amino acid metabolism may amplify monoamine imbalance, albeit with a small effect size [142].

Abnormalities in pre-psychotic hippocampus glutamate levels and increased dorsal striatal dopamine (DA) uptake interact [143]. Following the dopamine hypothesis, the excitation–inhibition imbalance hypothesis emerged, suggesting that the glutamatergic and gamma-aminobutyric acid (GABAergic) systems are dysregulated [144,145]. Recent extensive data indicate lower glutamate, GABA, and DA levels in cortical regions of schizophrenia patients, alongside elevated glutamate levels in the basal ganglia and thalamus [146]; however, disruption of glutamate metabolism is caused by multi-pathway interactions [147], involving shifts in multiple metabolic links that exacerbate oxidative stress.

Glutamate enters the synaptic cleft and activates ionotropic glutamate receptors (α-amino-3-hydroxy-5-methylisoxazole-4-propionic acid (AMPA), kainate, and NMDA) and metabotropic glutamate receptors (mGluRs), regulating the initiation and modulation of glutamatergic neurotransmission. A damaged glutamate network in schizophrenia patients has been confirmed, and disease mutations markedly increase postsynaptic genes involved in NMDA and AMPA receptor signaling pathways [148,149,150]. The NMDA receptor comprises subunits encoded by the GRIN1, GRIN2A, and GRIN2B genes, and it requires the binding of two different ligands to activate the ion channel [151]. Glutamate binds to a location on the GluNR2/3 subunits, whereas glycine, D-serine, kynurenic acid (KYNA), or mGluR3 agonists (NAAG) bind to the GluNR1 subunit’s modulatory binding site. The dysfunction of NMDARs is crucial to the excitatory–inhibitory imbalance in schizophrenia, and extensive research has demonstrated its association with cognitive impairment [152,153,154,155,156]. In schizophrenia, NMDAR subunits GluN1 [157] and GluN2A/B [152,158,159] are significantly downregulated in the PFC. Reduced levels of postsynaptic scaffolding proteins (GRIA3/4, ATP1A3, GNAQ) in the auditory cortex correlate positively with cognitive scores [160], and genetic variations in NMDAR-encoding genes have also been identified [161]. NMDAR function is regulated by the co-agonists D-serine and glycine, whose phosphorylation is controlled by multiple kinases. Numerous studies have identified insufficient D-serine and glycine production in schizophrenia, which correlates with psychotic symptoms [162,163,164]. High-dose D-serine administration has been shown to alleviate negative symptoms [165,166,167,168]. D-serine/D-alanine is metabolized by DAAO [169,170]. Over-expression and variants of the D-amino acid oxidase activator (*DAOA*, also known as G72) gene are associated with schizophrenia [171,172,173,174,175]. *DAOA* inhibitors are considered novel therapeutic targets [176,177] for treating impaired D-serine metabolism [178]. Research indicates that mutations in the glycine cleavage system (GCS) genes may lead to schizophrenia-like symptoms [179]. Glycine Transporter 1 (GlyT1) inhibitors (carnosine [180], BI-RG7118 [181], BI-425809 [182]) boost synaptic glycine and NMDAR activity, alleviating negative symptoms. GlyT1 has emerged as a core therapeutic target [183]. Additionally, schizophrenia patients exhibit mGluR5 dysregulation [184], decreased mGluR5 activity, and mGluR2/3 agonists (LY2140023 [185], LY379268 [186], LY395756 [187]) restore NMDAR/GABAAR balance and improve cognition. The endogenous mGluR3 ligand NAAG inhibits glutamate, GABA, and glycine release [188], with the schizophrenic brain showing lower NAAG levels [102,189]. D-aspartic acid (d-Asp) activates NMDARs via the GluN2 site and promotes glutamate release [190]. Postmortem studies reveal significantly reduced d-Asp levels in the prefrontal cortex of schizophrenia patients [191], and d-Asp supplementation antagonizes PCP-induced schizophrenia-like behaviors [192]. In summary, the glutamatergic dysregulation in schizophrenia may not stem from a single receptor defect but rather involve abnormalities across multiple pathways, and the above evidence provides a theoretical basis for precise interventions.

The cystine/glutamate antiporter (xc^−^ system) comprises a heavy chain subunit (4F2hc, SLC3A2) and a light chain subunit (xCT, SLC7A11), exchanging extracellular cystine for intracellular glutamate. This process regulates synaptic glutamate levels while providing the rate-limiting substrate for the synthesis of glutathione (GSH), an antioxidant [193]. xc^−^ system dysfunction is implicated in schizophrenia pathogenesis [194]. Excessive competition for extracellular glutamate inhibits intracellular cystine uptake, triggering oxidative stress-related cell death. One study shows reduced peripheral leukocyte mRNA expression of SLC3A2 and SLC7A11 [195]; another shows elevated xc^ protein levels in the dorsolateral prefrontal cortex [196]. However, only two studies have reported these findings, necessitating further research to confirm these results. Concurrently, lower EAAT1/2 expression and polymorphisms in schizophrenia patients limit glutamate clearance, which correlates with illness severity and cognitive deficits [197,198,199,200,201]. Abnormal splicing variants of EAAT1/2 are also observed in the anterior cingulate cortex [202]. The evidence suggests that glutamate clearance is equally important, offering a novel therapeutic strategy for schizophrenia.

Astrocytes absorb glutamate, convert it into glutamine (Gln) via glutamine synthetase (GS), and then transport it to neurons, where it is hydrolyzed into N-acetylaspartate (NAA) and glutamate—this constitutes the glutamate–glutamine cycle (Glx cycle). NAA serves as a marker of neuronal integrity. Schizophrenia is characterized by widespread impairment of Glx cycle homeostasis. Some studies have identified reduced NAA/Cr and Glx/Cr ratios in the hippocampus and DLPFC of schizophrenia patients [203,204], while others have demonstrated elevated Gln/Glu ratios in early-stage, untreated patients [205], potentially linked to neuronal degeneration. However, as the disease progresses, Glu, Gln, and NAA levels decrease significantly across various brain regions (frontal cortex, hippocampus, thalamus) in chronic patients [206]. Both clinical patients and animal models exhibit decreased Glu and increased Gln in PFC. NAA correlates negatively with age and disease duration [207,208,209,210,211,212,213], though isolated studies report elevated NAA in the hippocampal region of chronic patients [214] and the prefrontal cortex of high-risk adolescents [215]. Individual variation in damage to neurons may correlate with gray-white matter discrepancies [216]. Furthermore, peripheral blood 2-AG and ACC Glx levels in schizophrenia spectrum patients were negatively correlated, indicating that the ECS system may interact with the amino acid-lipid metabolism in disease mechanisms [217]. Collectively, Glx metabolic dysfunction is a key cross-stage metabolic characteristic of schizophrenia, providing prospective targets for early detection and treatment.

Kynurenine, a terminal metabolite of tryptophan via the kynurenine (KYN) pathway, has antagonistic effects on both NMDA and α7nACh receptors [218]. Elevated kynurenine levels inhibit glutamatergic and cholinergic transmission, which correlates with cognitive impairments [219]. Proinflammatory cytokines and oxidative stress stimulate KYN pathway metabolism, which is initiated by indoleamine 2,3-dioxygenase (IDO)/tryptophan 2,3-dioxygenase (TDO) [220,221] and is regulated in three steps—hydroxylation by kynurenine monooxygenase (KMO) and transamination by kynurenine transaminase (KAT)—determining the KYNA–quinolinic acid (QUIN) balance. QUIN, an NMDA receptor agonist, aggravates immunological inflammation and oxidative stress [222]. Reduced KMO gene expression and enzyme activity are observed in the prefrontal cortex of schizophrenia patients [223,224], while elevated KYNA levels are found in cerebrospinal fluid and postmortem brain tissue [223,225,226,227,228,229]. However, the results of KYNA in peripheral tissues (plasma, saliva, and skin fibroblasts) are discordant [230,231,232,233]. Conversely, 3-HK and QUINA are frequently disrupted in schizophrenic brains [234], while serum 3-HK levels show elevated [233], decreased [231,235], or unchanged [236] patterns, I believe these inconsistent findings may relate to factors such as the site of detection and disease severity [237]. Animal studies simultaneously demonstrate the interplay among these three components: knocking out the KMO gene or inhibiting the IDO/TDO/KMO pathways increases KYNA, decreases 3-HK, and alleviates oxidative stress and schizophrenia symptoms [238,239]. Proinflammatory factors (e.g., IL-6) have been shown to activate the IDO/KMO bypass, with studies demonstrating that this also increases KYNA production and significantly exacerbates schizophrenia symptoms [240,241,242], forming a pathological feedback loop. In summary, KYNA/QUINA imbalance is an important component of the glutamatergic hypothesis in schizophrenia. Targeting KMO, IDO/TDO, or α7nACh receptors for orthosteric regulation can restore KYNA–QUINA balance, providing new anti-inflammatory and cognitive-enhancing therapies.

Glutamate is converted into GABA via glutamate decarboxylase (GAD), thereby inhibiting glutamatergic excitatory signaling. Reduced GABAergic interneurons in the prefrontal cortex and decreased GAD mRNA expression are observed in schizophrenia [243,244]. The research indicates this leads to decreased GABA/Cr levels, which are positively connected with cognitive impairment. There is also evidence of decreased Glu and NAA in the prefrontal cortex and decreased GABA in the parieto-occipital region, with more noticeable effects in males [245]. Reduced GABA-A/BZ receptor binding in the right caudate nucleus occurs during early disease onset in persons at high risk for schizophrenia (UHR) [246]. GSH, an antioxidant, is generated from glutamate, cysteine, and glycine by gamma-glutamylcysteinyl synthase (GCL). The GCL gene and its variations increase vulnerability to schizophrenia [247,248], and impaired early GSH synthesis may trigger schizophrenia-like behaviors in adulthood [249]. Research indicates schizophrenic patients exhibit persistently reduced GSH levels in the brain and elevated NO and MDA concentrations [250]. In chronic patients, GSH levels in cerebrospinal fluid and the medial prefrontal cortex correlate inversely with negative symptom severity [251]. Multiple animal model studies have demonstrated that supplementation with glutathione precursors (such as N-acetylcysteine (NAC) [252,253,254,255,256,257,258,259] or alpha-lipoic acid (ALA) [260]) can reverse schizophrenia-like symptoms, oxidative stress, and neurotransmitter imbalances. In summary, GABA depletion and GSH antioxidant deficiency contribute to glutamatergic hyperexcitability and oxidative damage. In particular, GSH is frequently targeted as an intervention for oxidative stress in schizophrenia.

A meta-analysis examining the relationship between glutamate and brain metabolites in schizophrenia within recent magnetic resonance spectroscopy (MRS) studies suggests that alterations in glutamate levels may stem from metabolic impairment at the mitochondrial level [261]. Astrocytes regulate glutamatergic activity by clearing synaptic glutamate, maintaining glutamate-glutamine cycling, and initiating mitochondrial catabolism via glutamate dehydrogenase [262]. This process is closely associated with peripheral redox imbalances and energy metabolism disorders [263].

Based on the above evidence, amino acid metabolism abnormalities in schizophrenia persist throughout its development, with astrocytes serving as a critical site. Influenced by inflammation and energy metabolism, these abnormalities primarily contribute to imbalances in the dopamine (DA), glutamate (Glu), and gamma-aminobutyric acid (GABA) systems, as well as oxidative stress (Table 3). Nevertheless, they offer numerous effective targets for clinical intervention.

### 2.4. Schizophrenia and Abnormal Nucleotide Metabolism

Nucleotide metabolism is the central center for DNA/RNA synthesis and repair. Within this framework, one-carbon (C1) metabolism—encompassing DNA methylation (methionine) and homocysteine metabolism—acts as an interface between diverse pathogenic factors in schizophrenia, such as abnormal monoamine transmission, epigenetic dysregulation, oxidative stress, and maternal hyperhomocysteinemia [264]. Studies have observed insufficient levels of C1 molecules in the serum of schizophrenia patients [265]. Given that folate and vitamin B12 participate in DNA methylation and repair processes [266], one study found that abnormal DNA methylation in schizophrenia patients affects the amount of specific NMDAR subunits, lowering overall NMDAR activity [267]. Enzymes involved in C1 metabolism include methylenetetrahydrofolate reductase (MTHFR) [268,269] and nicotinamide N-methyltransferase (NNMT) [270], whose gene polymorphisms are associated with schizophrenia. The MTHFR C677T polymorphism, for instance, is linked to reduced total DNA methylation levels in female schizophrenia patients [271,272]. Hyperhomocysteinemia may increase the risk of schizophrenia [271,273], and multiple studies consistently demonstrate that schizophrenia patients have lower plasma folate, vitamin B12, and pyridoxal phosphate levels [274,275,276], as well as higher homocysteine levels [277,278,279]. In first-episode patients, low folate and high Hcy levels are associated with negative symptoms and cognitive deficits, with more pronounced effects in younger patients [280,281]. Furthermore, studies have revealed a positive correlation between plasma total homocysteine levels at a specific CpG site and DNA methylation [282], contradicting the previously observed pattern of widespread hypomethylation. This discrepancy may be related to medication effects or the specific testing site.

Mitochondrial dysfunction may result in DNA damage and oxidative stress, which affects nucleotide metabolism. Single nucleotide polymorphisms (SNPs) in mitochondrial DNA (mtDNA), such as the NADH oxidase gene, increase the risk of schizophrenia [283]. Studies have revealed enhanced purine degradation in schizophrenia, with significantly elevated xanthine (XAN) concentrations in patient plasma [235], alongside disturbances in nucleotide precursors like glutamate, arginine, and ornithine [284]. I hypothesize these may all relate to mitochondrial energy imbalance. Hyperhomocysteinemia, folate insufficiency, and mtDNA damage all contribute to oxidative stress, which suppresses C1 enzyme activity and mitochondrial respiration [21] (Table 4), thereby forming a vicious cycle of “hypomethylation–oxidative stress–energy crisis.”

**Figure 1:**
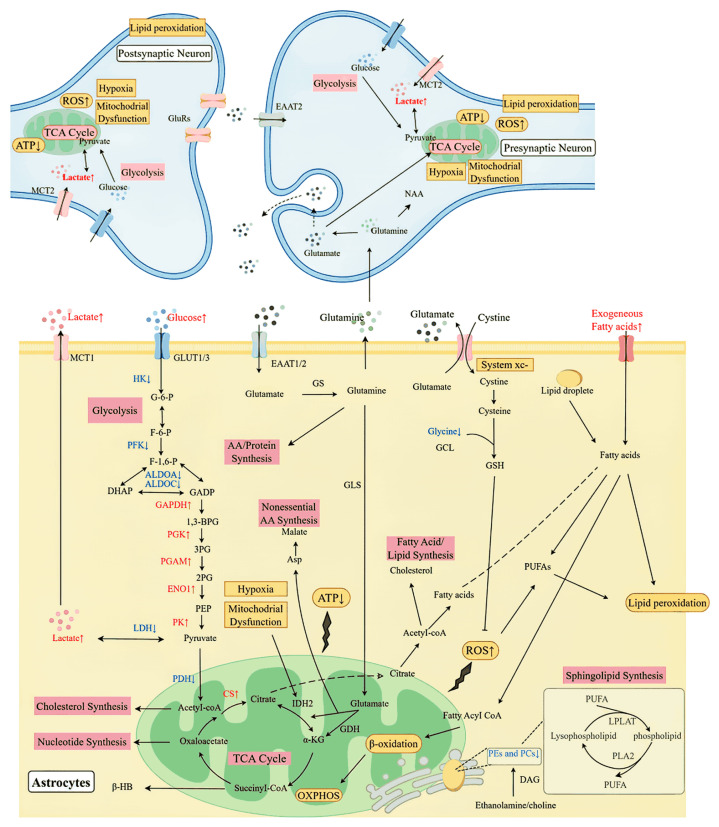
Abnormal metabolic profile in neurons and astrocytes of schizophrenia. Abnormal glycolysis pathway, impaired TCA cycle function, lactic acid accumulation; abnormal lipid metabolism (increased exogenous fatty acid uptake, lipid peroxidation, disrupted sphingolipid synthesis), disrupted lipid synthesis; disrupted glutamate–glutamine cycle, insufficient GSH synthesis. Blue arrow: decrease; Red arrow: increase; The dots represent metabolites: lactic acid (red), glucose (blue), glutamine (green), glutamic acid (purple); The yellow box indicates abnormalities. This figure was created by Figdraw2.0, https://www.figdraw.com.

In summary, various metabolic mechanisms interact to contribute to the energy metabolism disorders, oxidative stress, and neurotransmitter metabolism abnormalities observed in psychiatric patients, thereby driving disease progression. Targeting abnormalities in these metabolic pathways may influence disease phenotype and severity, suggesting that metabolic mechanisms represent important therapeutic targets for schizophrenia. The primary metabolic abnormalities in schizophrenia are shown in Figure 1 by figdraw.

## 3. Electroconvulsive Therapy for Schizophrenia

Electroconvulsive therapy (ECT) was originally developed in 1934. It is a safe and effective physical therapy for schizophrenia that works by stimulating the brain with electrical currents to elicit therapeutic generalized convulsive activity. It has been proven to rapidly improve both positive and negative symptoms, playing a crucial role particularly in treatment-resistant schizophrenia [285,286]. Initially, four primary theories were proposed to explain ECT’s mechanism of action: the traditional monoamine neurotransmitter hypothesis, the neuroendocrine theory, the anticonvulsant theory, and the neurotrophic theory. Based on the neurofunctional, endocrine, and immunological changes reported after ECT treatment for psychiatric diseases, researchers summarized that ECT’s therapeutic mechanism is related to related systems [287]. However, these systems do not appear to act independently; rather, they synergistically contribute to the therapeutic effect.

### 3.1. ECT Functions Through the Coordinated Interaction Between Different Systems

#### 3.1.1. Nervous System

Reduced neurogenesis in many regions of the brain has been linked to psychiatric disorders [288]. Research reveals that genes related to ECT efficacy are predominantly enriched in neurotrophic factor, mitogen-activated protein kinase, and long-term potentiation signaling pathways [289], suggesting ECT may exert antipsychotic effects by promoting neurogenesis and normalizing multiple neurotransmitter functions. In clinical trials, ECT is frequently utilized as an augmentation technique for clozapine-resistant schizophrenia, enhancing dopamine D2 receptor efficacy and improving clinical symptoms [140,290,291]. However, the precise position within the dopamine system where this action occurs is unknown. Impaired glutamatergic neurotransmission in schizophrenia patients has long been proven. Previous research on ECT’s effects on glutamate was limited to depression and Alzheimer’s disease [292,293,294]. Recent research reveals that ECT treatment (4 weeks) can restore GABA concentration in the prefrontal cortex of schizophrenia patients, an effect which was not observed in the pharmacologically treated group [295]. However, its efficacy in elevating neurotransmitter levels showed no difference compared to the pure medication group. ECT also upregulates serum BDNF concentration in treatment-resistant schizophrenia patients and exhibits a negative correlation with PANSS scores, suggesting potential mediation of synaptic remodeling via neurotrophic factors [296]. Furthermore, following ECT treatment, schizophrenia patients exhibited increased whole-brain gray matter volume (GMV) in the bilateral parahippocampal gyrus/hippocampus, right middle/superior temporal gyrus, and right insula. Notably, right parahippocampal gyrus/ hippocampus GMV changes showed a significant positive correlation with reduced PANSS positive subscale scores, suggesting ECT may improve positive symptoms by targeting limbic brain regions [297]. This is consistent with previous findings that ECT promotes neurogenesis in these areas. Numerous studies have identified abnormal brain functional networks in schizophrenia patients, which can be normalized with ECT. These include the default mode network (DMN) [298,299,300], prefrontal cortical networks, the hippocampus [301], and the cerebellum [302]. The extent of these changes correlates with treatment outcomes [303,304,305]. Because most of these studies are single-arm, further replicated trials are required to confirm ECT’s effects on the schizophrenic neurological system.

#### 3.1.2. Immune System

Postmortem studies on schizophrenia demonstrate abnormalities in the number of neurons and glial cells. The glial cell hypothesis of central inflammation and the neuroinflammation-related neurogenesis hypothesis propose that schizophrenia arises from the hyperactivity of microglia and astrocytes [306]. Part of ECT’s therapeutic effect may relate to its action on glial cells [307]. One study found that in a schizophrenia animal model mouse, the gene expression levels of CD11b in microglia within the dentate gyrus and CA1/CA3 regions, as well as GFAP expression in astrocytes within the GD and CA1 regions, were higher than in the control group. However, these levels decreased after repeated ECT treatment, indicating that both microglia and astrocyte activity were inhibited [308,309]. Another study demonstrated that in a neurodevelopmental animal model of schizophrenia, repeated ECT treatment improved MK-801-induced pre-pulse inhibition deficits. mRNA sequencing and qPCR analysis of the prefrontal cortex revealed that Egr1, Mmp9, and S100a6 were the central genes, while the interleukin-17 (IL-17), nuclear factor κB (NF-κB), and tumor necrosis factor (TNF) signaling pathways were determined to be the three most relevant pathways [310]. These findings suggest ECT directly suppresses glial hyperreactivity and blocks pro-inflammatory pathways. However, all of these studies were conducted in animal models with small sample sizes, necessitating urgent clinical investigations.

ECT therapy rapidly induces systemic anti-inflammatory effects in schizophrenia. Within two hours of a single ECT treatment, peripheral white blood cells, neutrophils, and lymphocytes decrease in schizophrenia patients [311]. Additionally, Nitric oxide synthase (iNOS), nitrite, and prostaglandin E-2 (PGE2), which are downstream of the nuclear factor κB (NF-κB) pathway, decrease [312]. Multiple ECT sessions demonstrate selective pro-inflammatory/anti-inflammatory rebalancing. Patients with treatment-resistant schizophrenia exhibit lower baseline serum transforming growth factor-β (TGF-β) and higher NF-κB levels, with no significant differences in IL-4 or myeloperoxidase (MPO) concentrations. After nine ECT sessions, TGF-β and IL-4 levels significantly increased alongside clinical improvement, while NF-κB levels were elevated. The concentrations of IL-4 and myeloperoxidase (MPO) were not significantly different. After 9 ECT sessions, TGF-β and IL-4 levels increased and accompanied clinical improvement, whereas MPO and NF-κB activation remained unchanged [313]. After 10 ECT treatments, a gradual decrease in TNF-α was observed [314]. Matrix metalloproteinase-9 (MMP-9) is specifically associated with glutamatergic signaling and regulation of hippocampal neuroplasticity. Peripheral blood MMP-9 levels are typically elevated in schizophrenia patients and positively correlated with negative symptoms [315]. One study found no significant difference in baseline MMP-9 levels between schizophrenia patients and controls, but MMP-9 levels decreased significantly after 10 ECT sessions, though this was unrelated to symptom severity [316]. Proinflammatory substances have been demonstrated to influence NMDAR function by stimulating IDO, a key enzyme in the kynurenine pathway [221]. In one study, patients were separated into high- and low-inflammation groups, and the low-inflammation group showed more clinical improvement. IL-18 mRNA levels decrease significantly after ECT, although KYN/TRP, KYNA/KYN, and IL-18 levels decreased only in the low-inflammation group. Cytokine levels were significantly correlated with KP metabolites, and baseline KYNA/TRP and IL-18 levels positively correlated with negative symptoms after ECT. These findings further demonstrate that the ECT-induced reduction in inflammation and its relationship with KP metabolites correlate with clinical efficacy [9].

Vascular endothelial growth factor (VEGF) plays an important role in angiogenesis and blood–brain barrier permeability. Studies have shown that treatment-resistant schizophrenia patients have lower baseline serum VEGF levels, with significant increases post-ECT treatment that are negatively correlated with PANSS scores [317]. This suggests ECT may protect neurons by enhancing angiogenesis, reshaping blood–brain barrier integrity, and reducing the penetration of peripheral inflammatory factors into the central nervous system.

#### 3.1.3. Endocrine System

Schizophrenia patients exhibit hyperactivity of the hypothalamic–pituitary–adrenal (HPA) axis, primarily maintaining elevated cortisol levels [318]. This hyperactivity contributes to multiple neurological alterations observed in these patients [319,320]. Chronic low-grade inflammation is commonly present in schizophrenia, and ECT may indirectly modulate immune responses by rapidly reducing HPA axis overactivation [321]. In one study, serum growth hormone (GH) levels decreased immediately during the fourth and eighth bilateral ECT sessions in schizophrenia patients, with no differences observed in subsequent sessions [322]. These initial serum GH alterations may indicate a dopamine-mediated ECT response. Further research suggests that after the first session of ECT treatment, schizophrenia patients show immediate increases in prolactin and a decrease in TSH [323]. It is hypothesized that ECT may reduce the free fraction of T4. Considering that elevated levels of this hormone have been reported in schizophrenia patients and those with suicidal ideation, this could have therapeutic implications. In summary, research on ECT’s effects on the endocrine system in schizophrenia remains limited, and its impact on the hypothalamic–pituitary–thyroid (HPT) axis remains controversial [324,325].

Collectively, ECT may influence schizophrenia through synergistic interactions across interconnected systems; however, variations observed in these studies primarily focus on clinical responses rather than neurobiological pathways [326]. It has been proposed that ECT increases GABA levels to reduce neuronal activity in schizophrenia patients, aligning with the monoamine hypothesis [293]. It may also relate to neurogenesis, as hypothalamic axis dysregulation leads to reduced brain volume in psychiatric patients. Depression patients can counteract this effect by regulating cortisol levels through ECT, which may similarly apply to comorbid schizophrenia patients. Furthermore, inflammation is associated with HPA and neurogenesis disruption. ECT can counteract these abnormalities by reducing IL-6 and TNF-α levels along with polymorphonuclear cell counts while increasing IL-4, TGF-β, and total leukocyte and lymphocyte percentages [311,313]. In summary, other neurochemical and neuroendocrine alterations following ECT are secondary phenomena associated with clinical improvement and the remodeling of dysfunctional networks (Table 5).

### 3.2. Relationship Between Metabolic Mechanisms and Various Systems in ECT Treatment for Schizophrenia and Its Correlation with Symptom Improvement

The effects of ECT on cerebral cortical blood flow [327] and metabolism have long been a focal point in molecular psychiatry research. Preliminary evidence suggests that ECT may participate in the pathophysiological processes of schizophrenia by regulating neurogenesis and neurotransmitter systems [327,328,329,330], although its precise molecular mechanisms remain to be fully elucidated. We searched the literature focusing on the relationship between metabolism and ECT in schizophrenia patients. Given the close association between glucose metabolism and brain energy homeostasis, we also included studies exploring changes in brain energy metabolites following ECT. Our findings suggest that metabolic mechanisms may serve as the pivotal link connecting ECT to its multifaceted systemic effects in schizophrenia. The transient, controlled physiological stress induced by ECT may trigger systemic “metabolic reset,” involving alterations in energy substrate utilization patterns and the generation of specific metabolic products. However, direct evidence for longitudinal causal relationships regarding how this metabolic reprogramming regulates neural circuitry, immune-inflammatory responses, and neuroendocrine axis function through specific molecular pathways remains lacking.

Current research on the relationship between metabolism and brain energy homeostasis in schizophrenia patients following ECT treatment is limited and varies in methodology. An observational study involving 99 patients (including those with depression, bipolar disorder, and schizophrenia) reported acute increases in blood glucose and total cholesterol levels after ECT [331], but the long-term metabolic effects, disease-specific differences, and clinical relevance remain unclear. A recent study employing comprehensive metabolomics analyzed plasma samples from schizophrenia patients (*n* = 78). Compared to controls, 542 metabolites exhibited differential expression (420 downregulated, 122 upregulated), primarily involving lipids in energy metabolism pathways. Following ECT treatment, 200 metabolites associated with glycolysis, ketone metabolism, and inflammatory pathways showed significant alterations (153 upregulated, 47 downregulated). Furthermore, the study identified 10 baseline metabolites capable of distinguishing ECT responders from non-responders. In responders, TRPV1/TRPA1 channel agonists (hydroxy-α-pipralid and piperine) associated with neuroprotection and inflammatory regulation were significantly elevated, indicating that the therapeutic efficacy of electroconvulsive therapy indeed involves metabolic reprogramming and inflammatory responses [332]. Under normal conditions, cellular enzymatic and non-enzymatic antioxidant defenses eliminate reactive oxygen species (ROS), which are metabolic byproducts; otherwise, they induce oxidative stress. Nearly all metabolic abnormalities in schizophrenia are accompanied by oxidative stress [333]. Redox reactions serve as a crossroads for multiple critical biochemical pathways, including mitochondrial function, immune signaling, and neuroplasticity, and are closely linked to cognitive function [334]. One study found that baseline serum malondialdehyde (MDA), catalase (CAT), and NO levels were higher in schizophrenia patients (*n* = 28) than in controls (*n* = 20). After nine ECT sessions, only serum MDA levels significantly decreased, accompanied by improvements in BPRS, SANS, and SAPS scores—with more pronounced changes in first-episode patients [335], suggesting ECT may selectively modulate oxidative stress markers. However, this study lacked randomization, had a limited sample size, and failed to control for antipsychotic medication use as a confounding factor. Additionally, ECT elevates serum BDNF levels, correlating with changes in psychotic symptoms [296]. Based on existing evidence, we hypothesize that ECT may influence cognitive function by modulating the interaction between oxidative stress and BDNF [336], though this causal chain requires validation under rigorous experimental conditions. Notably, oligodendrocyte injury and subsequent white matter abnormalities are considered key pathological underpinnings of cognitive impairment in schizophrenia [337]. However, current ECT research has focused solely on the relationship between gray matter damage and positive symptoms [297], limiting our understanding of ECT’s comprehensive neuroprotective effects. Furthermore, studies on ECT’s metabolic effects show inconsistencies across disease stages. Some research suggests first-episode patients may exhibit more pronounced improvements in oxidative stress markers than chronic patients [335], while other studies using magnetic resonance spectroscopy (MRS) indicate chronic patients may also demonstrate positive changes in neuronal metabolic markers. One study showed that after 8 sessions of modified ECT, schizophrenia patients (*n* = 31, including first-episode and chronic patients) exhibited significantly increased NAA/Cr ratios in the left prefrontal cortex and thalamus, with this change correlated with age at onset, disease duration, and baseline severity [338]. However, the absence of a control group in this study limits the reliability of causal inferences. Another controlled study using MRS to assess brain metabolites in chronic schizophrenia patients (*n* = 10) found that the ECT–combination therapy group exhibited elevated NAA/Cr ratios and reduced choline (Cho)/Cr ratios in the left prefrontal cortex, suggesting potential improvements in neuronal integrity and reduced cell membrane turnover [339,340]. However, this study had an extremely small sample size, a short follow-up period (4 weeks), and was not randomized.

Recent studies indicate that the therapeutic effects of ECT involve inflammatory responses [332], with existing ECT-related research suggesting this may be linked to shifts in immune cell metabolic patterns. Research using electroconvulsive stimulation (ECS) animal models demonstrates that repeated ECS promotes a metabolic shift in central microglia and peripheral immune cells from a glycolysis-dominant pro-inflammatory mode to an oxidative phosphorylation-dominant anti-inflammatory mode [310,311,312,313,314]. This finding provides a theoretical framework for explaining the post-ECT reduction in pro-inflammatory cytokines (e.g., IL-6, TNF-α) and the increase in anti-inflammatory factors (e.g., IL-10) [310,311,312,313,314]. However, it is crucial to emphasize that the aforementioned metabolic conversion mechanism requires direct validation in schizophrenia patients. An observational study found that peripheral blood levels of cytokines including IL-6, TNF-α, and NF-κB decreased in schizophrenia patients receiving ECT, while MMP-9 significantly decreased after 10 ECT sessions, though this was unrelated to symptom severity [316]. The MMP9/RAGE pathway is considered a key substrate for the interaction between oxidative stress and neuroinflammation [306,341]. Given that this study was based on a single cohort with a limited sample size (*n* = 21), the potential value of MMP-9 as a therapeutic target for ECT requires validation in larger, replicated studies. Furthermore, ECS can reduce microglial hyperactivation [308,309], and it reduces inflammation and its association with KP metabolites [9]. Low inflammation and tryptophan metabolism correlate with clinical efficacy. Cross-sectional association studies indicate co-regulation of kynurenine and the ECS system in schizophrenia, sharing common pathophysiological foundations in astrocyte distribution, inflammatory regulation, and neurotransmitter balance [342,343]. We hypothesize that the ECS may also be a therapeutic target for ECT, though this requires validation through interventional studies.

Schizophrenia patients frequently have persistent low-grade inflammation and hyperactivity of the HPA axis. Preliminary clinical observations indicate that ECT can quickly alleviate excessive activation of the HPA axis [321]. However, these findings only reflect acute endocrine responses during treatment and do not allow inference about whether ECT achieves long-term metabolic improvement through sustained reset of HPA axis function. Regarding the epigenetic effects of ECT, a microarray study identified differences in miRNA expression profiles before and after ECT treatment in schizophrenia patients (e.g., a downregulation trend of miR-20a-5p). However, the statistical power was insufficient, and no direct correlation with clinical symptom improvement was established [344].

Preliminary evidence from structural and functional neuroimaging studies consistently suggests that ECT’s efficacy in schizophrenia may partly stem from its regulatory effects on abnormal functional connectivity in key brain regions [298,299,300,301,302,304]. The hippocampus and insula are consistently identified as core target areas for ECT-induced neuroplastic changes [345]. These structural alterations show statistical correlations with clinical symptom improvement, suggesting that ECT may exert its antipsychotic effects by repairing neural circuit dysfunction in these regions. However, these studies are predominantly small-sample, non-randomized designs lacking long-term follow-up data. Some speculate that ECT’s efficacy may partly relate to its effects on glial cells [307], which play critical roles in various metabolic pathways associated with schizophrenia (e.g., energy metabolism, tryptophan metabolism, cytokines) [346]. These metabolites may regulate neuro-immune–endocrine interaction networks. Recent studies have validated the link between metabolic reprogramming (glycolysis, ketone metabolism) and ECT efficacy [332], though causal relationships require further validation through large-scale studies. Future studies should employ longitudinal multi-omics designs (combining metabolomics, lipidomics, and immune phenotyping) to analyze dynamic changes in metabolite profiles across different tissues of schizophrenia patients undergoing ECT. Correlating these with clinical efficacy, neuroimaging, and immune markers will deepen our understanding of ECT’s mechanisms of action. Furthermore, targeted metabolic interventions in animal models (e.g., specific diets or enzyme inhibitors) can directly validate whether certain key metabolic pathways are essential for ECT efficacy. Identifying baseline metabolic biomarkers associated with treatment response will facilitate the future precision targeting of ECT therapy.

### 3.3. Side Effects of ECT

Treatment resistance is the most common response to ECT, and some patients exhibit a poor response to ECT, potentially related to individual genetic background or immune characteristics. The primary adverse effect of ECT is cognitive impairment, though such side effects are typically mild and transient. In fact, numerous studies indicate that ECT does not impair cognitive function [347,348] and may even improve it [349,350]. While ECT may cause temporary memory impairment, its long-term neuroplastic effects partially offset these negative impacts. Furthermore, the adverse cognitive effects of ECT appear to depend on multiple factors, including the patient’s baseline cognitive status and potential cognitive reserve prior to treatment, as well as certain ECT parameters such as bilateral electrode placement, current intensity, and stimulation type.

## 4. Metabolic Changes in Other Physical Therapies for Schizophrenia

In addition to electroconvulsive therapy, other commonly used physical therapies for schizophrenia include transcranial magnetic stimulation (TMS), transcranial direct current stimulation (tDCS), and deep brain stimulation (DBS).

Both repetitive transcranial magnetic stimulation (rTMS) and transcranial direct current stimulation (tDCS) improve general psychotic symptoms, cognitive deficits, and anhedonia in schizophrenia [351,352,353,354], with mechanisms connected to brain network normalization [355]. Nevertheless, there is still a dearth of research on related metabolic alterations. Negative symptoms decreased after 4 weeks of rTMS treatment in chronic schizophrenia patients (*n* = 86) using oral paliperidone, and serum BDNF concentrations increased with stimulation duration [356], suggesting rTMS may stimulate BDNF synthesis to promote neuroplasticity. Schizophrenia patients who use different TMS modalities lose weight. Continuous theta burst stimulation (cTBS) decreased body weight and BMI in overweight schizophrenia patients [357], while 10 Hz rTMS (4 weeks) significantly reduced body weight in chronic patients [358]. Furthermore, improvements in body weight among patients undergoing iTBS while taking antipsychotic medication are correlated with factors including brain activity and the metabolism of glucose and lipids. TMS could improve symptoms by intervening with metabolic pathways since changes in appetite are linked to changes in brain structure, perfusion, function, cognitive control, microbiome, and neuroendocrine regulatory factors [359,360].

Low-frequency DBS to the basal ganglia reverses cognitive impairment induced by NMDA antagonist treatment in non-human primate models [361]. Clinical studies indicate that DBS modulates local and distant glucose metabolism in the anterior cingulate cortex (sgACC) or nucleus accumbens (NAc) of treatment-resistant schizophrenia patients, with clinical improvement associated with increased metabolic activity across extensive brain regions [362], suggesting that DBS may produce therapeutic effects by regulating brain metabolism.

## 5. The Relationship Between Antipsychotic Drugs and Metabolism in Schizophrenia Patients

The use of traditional antipsychotic drugs alone for treating schizophrenia often induces an independent “drug-induced” metabolic syndrome [8], which shares a common genetic basis with type 2 diabetes [363]. Long-term antipsychotic medication significantly increases body weight, BMI, blood lipids, and blood glucose levels [364,365], leading to impaired antioxidant defense systems [366] and heightened susceptibility to oxidative stress [367,368]. However, some antipsychotic drugs can improve metabolic abnormalities associated with schizophrenia (Table 6). Clozapine inhibits glucose uptake and glycolysis, shifts mitochondrial citrate into the cytoplasm to promote lipogenesis, and simultaneously reduces Akt pathway activation. These changes may prevent energy deficiency [369]. Treatment for 4 weeks also causes a short-term elevation in plasma IL-6 levels [370]. Haloperidol may inhibit glutathione peroxidase, disrupting neuronal redox homeostasis [371], and excessive use impairs dopamine release and AKT/GSK-3 signaling [372]. Risperidone monotherapy for 12 weeks raised serum SOD and CAT activity while decreasing MDA and GPx activity in patients; baseline CAT levels correlated with body weight or BMI [373], and oxidative stress markers predicted response to risperidone treatment [374]. Four weeks of olanzapine treatment similarly induced dyslipidemia characterized by elevated TG, TC, and LDL-C levels [375]. Furthermore, antipsychotic-induced constipation correlates with distinct lipid metabolism pathway dysregulation [376].

Antipsychotics may also have an influence on epigenetic mechanisms, with haloperidol consistently related to increased global hypermethylation and clozapine promoting hypomethylation across the epigenome [377]. Risperidone monotherapy normalizes DNA methylation patterns in schizophrenia patients, which correlate with clinical phenotype improvement [378], while also reducing serum homocysteine levels, suggesting one-carbon metabolism involvement [278]. Both risperidone and quetiapine regulate histone marks across distinct brain regions and cell types [379]. Furthermore, olanzapine may improve cognitive impairment by upregulating GluR1-Ser845 phosphorylation status [380].

**Table 6:** Effects of antipsychotic drugs on abnormal metabolism in schizophrenia.

Methods	Subjects	Antipsychotic Drugs	Changes of Markers	Therapeutic Outcomes	Refs.
Cell Experiments	SCZ patients, peripheral blood cells (*n* = 10)	Clozapine	Inhibits glycolysis, increases lipid droplet	Reduces apoptosis	[369]
Animal Experiments	Ketamine-induced SCZ model rats, male	Haloperidol (0.1 mg/kg)	GPx activity ↓ in brain regions (cortex, hippocampus, striatum)	Oxidative stress, lipid peroxidation	[371]
Animal Experiments	Male Wistar rats	Haloperidol (0.75 mg/kg), 14 consecutive days	Striatal DA, HVA, GSK-3α/β ↓; pGSK-3β/GSK-3β ratio ↑	Dopamine release, GSK-3 signaling↓	[372]
Prospective Longitudinal Study	First-episode, untreated SCZ patients (*n* = 225)	Risperidone monotherapy, 12 weeks	Baseline: SOD, CAT ↑; GPx, MDA ↓; After 12 weeks: Weight gainers showed SOD ↑, MDA ↓	Baseline SOD and GPx activity (75 gainers vs. 150 non-gainers)	[373]
Prospective Longitudinal Study	First-episode, untreated SCZ patients (*n* = 354)	Risperidone monotherapy, 12 weeks	Baseline: SOD, CAT, TAS ↑; GPx activity, MDA ↓; After 12 weeks: GPx activity ↓, TAS ↑, MDA ↓	Smaller GPx activity decline/treatment response	[374]
Longitudinal Cohort	Treat-resistant SCZ patients (*n* = 38)	Clozapine initiation therapy, 6-month follow-up	Peripheral blood DNA methylation abnormalities; B-cell proportion initially decreased then increased	Methylation changes, clinical response	[377]
Longitudinal Cohort	First-episode, treat-naive SCZ Chinese patients (*n* = 38)	Risperidone monotherapy 4–6 mg/day, 8 weeks	Peripheral blood DNA methylation alterations at 5979 CpG sites	Methylation normalization, cognitive improvements	[378]
Randomized Controlled Trial	First-episode, untreated SCZ patients (*n* = 56)	Risperidone (3.84 ± 0.95 mg/day, 12 weeks)	Serum Hcy ↓	Negative symptom improvement	[278]
Animal Experiments	Adult male MK-801 model rats	Oligan (2 mg/kg), haloperidol (0.1 mg/kg) intraperitoneal injection for 3 days	Oligan group showed ↑ 60% hippocampal GluR1 Ser845 phosphorylation	Spatial memory/working memory deficits, hippocampal damage	[380]

Dopamine (DA), vanillic acid (HVA), Glycogen synthase kinase-3 (GSK), Superoxide dismutase (SOD), Catalase (CAT), Glutathione peroxidase (GPx), malondialdehyde (MDA), total antioxidant status (TAS), homocysteine (Hcy).

## 6. The Role of Metabolic Mechanisms in Other Psychiatric Disorders

Metabolic mechanisms also play a significant role in the onset and progression of psychiatric disorders such as major depressive disorder (MDD), bipolar disorder (BD), and obsessive–compulsive disorder (OCD). A systematic review revealed that MDD and BD exhibit 97 and 47 lipid alterations, respectively, with some overlap with schizophrenia, sharing genes like *ABCA13*, *DGKZ*, and *FADS*, as well as the “inflammation–lipid–mitochondria” pathway. Meanwhile, OCD has been linked to sphingolipid signaling and peroxisome metabolism [381].

There are abnormalities in multiple metabolic pathways of MDD, and different antidepressant treatments may improve depressed symptoms by regulating these metabolic pathways. Impaired glucose metabolism is a core component, manifesting as insulin resistance and dysregulation of the GLUT1/insulin signaling pathway [382,383,384], while impaired TCA cycle function and reduced ATP production contribute to heightened depressive symptoms [385,386]. Abnormal glucose metabolism in hippocampal regions is closely connected with anhedonia [387] and impaired cognition [388]. Extensive research demonstrates that abnormal glucose metabolism in MDD brain regions correlates with mitochondrial dysfunction [389,390,391], impaired brain tissue energy metabolism [392], and elevated oxidative stress [393,394]. Concurrently, the astrocyte–neuron lactate shuttle mechanism is impaired. Autopsy studies confirm significantly reduced astrocyte density in multiple brain regions, including the prefrontal cortex, orbitofrontal cortex, and dorsolateral prefrontal cortex [395,396], disrupting ATP and lactate production by astrocytes [397]. Furthermore, animal studies indicate that inhibiting astrocytic glycogen utilization [398,399] and ATP production induces depressive-like behaviors, whereas promoting endogenous ATP release produces antidepressant effects [400,401]. Common antidepressants such as norepinephrine and glucocorticoids regulate glycogen conversion by binding to receptors on astrocytes [402]. Lipid metabolism disorders also contribute to pathogenesis. MDD patients with abnormal blood glucose levels often exhibit concomitant lipid and thyroid hormone dysregulation, which correlates with clinical symptoms [403,404,405]. Most differentially expressed lipid metabolites show negative correlations with depression symptom scores [406]. MDD patients often exhibit downregulation of long-chain fatty acids, upregulation of lysophosphatidic acid and ceramides [407,408], and significantly reduced total cholesterol (TC) levels [409], potentially correlated with disease severity [410]. This identifies novel therapeutic targets for MDD. Most MDD patients exhibit abnormal glutamatergic signaling in the PFC, with decreased NAA, total choline (tCho), and total creatine (tCr) [411]. One study showed that depressed patients with normal ACTH exhibited elevated Glx and reduced GSH levels, which correlated with depressive symptoms and cognitive function [412]. Abnormal TRP–KYN metabolic pathways and their metabolites are also closely associated with MDD [413,414], and plasma tryptophan levels correlate with antidepressant treatment response [415]. Abnormalities in purine metabolism [416] and methylation of certain genes [417] are observed in MDD, involving oxidative stress-related genes (HACE1, SHANK2) [418], HPA axis-related genes [419], and the MTHFR genotype associated with homocysteine metabolism [420]. Furthermore, high HTR1B methylation interacts with the rs6298 AA/AG genotype to influence antidepressant efficacy [421].

Increased cerebral lactate [422], reduced cerebral pH [57], energy dysregulation, and mitochondrial dysfunction [423] constitute the core pathophysiological features of BD. Manic episodes may be characterized by an upsurge in glutamine metabolism [424]. Oxidative stress is also a major pathogenic mechanism in BD, with significantly decreased plasma GSH and total thiol levels, while malondialdehyde (MDA), advanced oxidation protein products (AOPP), protein carbonyl (PC), homocysteine (Hcys) concentrations, and glutathione peroxidase (GSH–Px) activity are markedly elevated [425]. The antioxidant genes SOD2 and GPX3 correlate with structural abnormalities in the prefrontal cortex of young BD patients [426]. Impaired lipid metabolism in BD is closely associated with circadian rhythm-driven alterations in lipid droplet homeostasis [427], with deregulation of arachidonic acid and other polyunsaturated fatty acid production possibly representing a pathogenic pathway [428]. Both schizophrenia spectrum disorders and BD exhibit dysfunction in kynurenine metabolism and the noradrenergic and purinergic systems [235]. Furthermore, partial genetic susceptibility to schizophrenia, bipolar disorder, and major depressive disorder correlates with placental DNA methylation [429,430], while increased methylation at CpG sites of BDNF alleles is associated with early-stage BD [431]. Lithium salts, commonly used as mood stabilizers, bind to various ATP-binding enzymes [432]. Long-term lithium treatment is associated with increased telomerase reverse transcriptase (TERT) expression [433], which may improve mitochondrial function and reduce oxidative stress.

NAA/Cr levels in the caudate nucleus of OCD patients are lower than those in healthy individuals and negatively correlated with oxidative stress markers [434]. This is frequently accompanied by neurolipid metabolism abnormalities such as lipid peroxidation, phospholipid metabolism disruption, and sphingolipid signaling impairment [381,435]. Meanwhile, KP metabolism in OCD may be influenced by oxidative stress and abnormal levels of the inflammatory mediator interferon-gamma (IFN-γ) [436].

## 7. Conclusions and Future Directions

Electroconvulsive therapy (ECT) is an effective physical treatment for schizophrenia, with complex, multiple mechanisms of action. This review analyzes various metabolic abnormalities in schizophrenia and illuminates the significance and promise of metabolic pathways in ECT treatment for schizophrenia, with the aim of providing a theoretical foundation and clinical advice for future studies in this field.

Overall, metabolic reprogramming may serve as the pivotal link connecting the multisystem effects of ECT treatment for schizophrenia with clinical symptom improvement: (1) ECT efficacy is primarily associated with energy metabolism pathways. Notably, baseline metabolic characteristics hold predictive value: specific metabolite levels, such as those of TRPV1/TRPA1 channel agonists, can distinguish ECT responders from non-responders. Additionally, ECT induces elevated NAA/Cr ratios and altered choline metabolism in patients, reflecting neuronal integrity restoration and slowed cell membrane turnover. These neuroimaging changes correlate significantly with improved clinical symptom scores, particularly in first-episode patients. (2) ECT selectively reduces serum MDA levels while elevating BDNF. This redox state may interact with neurotrophic factors to enhance cognitive function. (3) ECT induces peripheral and central immune cells to shift from pro-inflammatory glycolysis to anti-inflammatory oxidative phosphorylation metabolism. This manifests as decreased pro-inflammatory factors (e.g., IL-6, TNF-α) and increased anti-inflammatory factors (e.g., IL-10), accompanied by regulation of the tryptophan–kynurenine metabolic pathway. This metabolic–immune coupling change occurs synchronously with the remission of psychotic symptoms. However, current evidence is largely based on small-sample, non-randomized short-term observations lacking longitudinal causal validation. Caution is warranted against directly extrapolating animal data to clinical practice. Future research should employ multi-omics integration (metabolomics, lipidomics, immunophenotyping) combined with long-term neuroimaging follow-up to establish causal pathways linking metabolism, neural circuits, and clinical symptoms. This approach will enable the development of precision ECT treatment strategies based on metabolic phenotypes. Concurrently, investigating the impact of various ECT technical parameters—such as electrode placement, electrical wave magnitude and duration, anesthetic and muscle relaxant usage, and treatment frequency—is essential to further improve the safety and efficacy of ECT therapy.

In summary, the mechanism of ECT in treating schizophrenia is closely linked to metabolic pathways. Deepening our understanding of these metabolic mechanisms not only enhances our knowledge of schizophrenia’s pathophysiology but also may provide critical insights for building new medications or bettering existing treatments. Future research should continue to focus on screening metabolomic biomarkers, developing targeted intervention strategies, and deepening the exploration of ECT mechanisms. This will aim to provide more effective treatment options for schizophrenia patients, improving their prognosis and quality of life.

## Figures and Tables

**Table 1:** Glucose metabolism enzymes and lactate in schizophrenia.

Method	Subjects	Abnormal Indicators	Mechanisms/ Effects	Refs.
LCM-qPCR	SCZ patients (DLPFC)	PFK and HK activity ↓; mRNA: MCT1 ↑; GLUT1 and GLUT3 ↓	Abnormal glucose transport, insufficient energy	[26]
Genome-wide linkage analysis	NIMH SCZ genetics sample	PFKFB2, HK3, PK3	Abnormal glycolytic enzymes, energy metabolism imbalance	[31]
Proteomics analysis	SCZ postmortem (hippocampus)	gluconeogenesis pathways (ALDOC ↓, ENO1 ↑); glycolysis pathways (ALDOA, ALDOC, ENO2 ↓)	Abnormal glycolytic enzymes, energy metabolism imbalance	[32]
GC-MS	First-episode, untreated SCZ patients	11 to 13 glucose metabolites	Glycolysis and TCA cycle	[12]
Western blot	SCZ patients (caudate nucleus)	PDHB ↓	Abnormal glucose metabolism/energy metabolism	[33]
Spectrophotometry	SCZ parietal cortex (BA7)	G6PD activity and mitochondrial HK1 (negative correlation)	Glucose metabolism, mitochondrial dysfunction, oxidative stress	[34]
Whole-genome sequencing	SCZ patients	PFK, ACC ↓	Impaired intestinal glucose/lipid metabolism	[35]
High-resolution Respiratory measurement	MK-801 rat (prefrontal cortex, hippocampus, striatum)	MDH, CS ↑, LDH, HK ↓	Abnormal glucose metabolism/energy metabolism	[36]
Spectrometry	SCZ postmortem (prefrontal cortex, hippocampus)	Lac, G6PD, Phosphatidylcholine ↑	Enhanced glycolysis, impaired mitochondrial oxidative phosphorylation	[14]
Metabolomics	SCZ patients (ACC)	Blood Lac ↓	Energy metabolism	[7]
Multi-omics integration	SCZ patients	10 Key Glycolytic Enzymes ↓ (HK1, PDHA1, PKM, etc.)	Abnormal glucose metabolism, mitochondrial dysfunction, oxidative stress	[47]
Colorimetric method	SCZ Postmortem (DLPFC), *DISC1* mutant mice, GluN1 knockdown mice, iPSC-derived neurons	Postmortem brain tissue and iPSC-derived neurons Lac ↑; Astrocyte-specific *DISC1* mutant mice Lac ↓	Disruption of ANLS; mitochondrial dysfunction, energy metabolism imbalance	[49]
Proteomics analysis	SCZ patients	Lac and GLUT1 ↑, insulin receptor ↓ (peripheral monocytes); insulin ↑ (serum)	Insulin resistance, abnormal glycolytic pathway	[50]
Targeted metabolomics	SCZ patients (serum)	D-lactate ↑, L-lactate, 3-hydroxybutyric acid, glutathione precursors ↓	Oxidative stress, abnormal glycolytic pathway.	[51]
Biochemical analysis	SCZ patients	Lac ↑ (CSF)	Mitochondrial dysfunction, enhanced glycolysis	[52]
GC-MS	SCZ patients	Serum glucose and Lac ↑, 1,3-diphosphoglycerate ↓	Abnormal glucose metabolism oxidative stress	[53]
1H NMR spectroscopy	SCZ patients	Lac and pyruvate ↑, GABA ↓	Imbalance in the glycolysis–glutamate pathway	[54]
mtDNA Sequencing	SCZ patients	Blood lac ↑ during exercise	mtDNA mutation, OXPHOS defect	[55]
7 Tesla proton (1H)-MRS	SCZ patients	Lac ↑ in the anterior cingulate cortex	Impaired mitochondrial OXPHOS, abnormal glucose metabolism	[64]
Molecular biology	PND21-34 Isolated rats	Lac ↑ in frontal lobe	Impaired OXPHOS	[65]
Cell experiments	MK-801 Mouse (hippocampus)	Lac ↑, Pyruvate ↓	Abnormal glucose metabolism	[66]

Dorsolateral prefrontal cortex (DLPFC), Phosphofructokinase (PFK), Hexokinase (HK), Malate/lactate Transporter (MCT1), Glucose Transporter (GLUT), 6-Phosphofructokinase-2/ Fructose-2,6-bisphosphatase 2 (PFKFB2), Pyruvate Kinase 3 (PK3), Pyruvate Dehydrogenase Beta Subunit (PDHB), Glucose-6-phosphate dehydrogenase (G6PD), Acetyl-CoA carboxylase (ACC), Citrate synthase (CS), Malate dehydrogenase (MDH), Lactate dehydrogenase (LDH), cerebrospinal fluid (CSF), oxidative phosphorylation (OXPHOS), lactate (Lac), astrocyte-neuron lactate shuttle (ANLS).

**Table 2:** Lipid metabolism in the brains and surrounding tissues of SCZ patients.

Method	Subjects	Abnormal Indicators	Mechanisms/ Effects	Refs.
Targeted lipidomics	SCZ patients (Prefrontal cortex gray matter)	PC, PE, and phosphatidylserine levels ↓	Sphingolipid metabolism	[99]
Targeted lipidomics	SCZ patients (Prefrontal cortex gray matter)	NAPS, NAS ↑	Abnormal nerve membrane	[100]
Targeted lipidomics	SCZ patients (Prefrontal cortex gray matter)	Thioester, Phospholipid alcohol, NAPS ↑	impaired myelin structure	[101]
Targeted lipidomics	SCZ patients (Prefrontal cortex gray matter)	Sulfate ceramides, lactosyl ceramides, NAPS, and NAPEs ↑; Ceramides, NAEs, and NAAG ↓	Altered ceramide metabolism pathways	[102]
31P-MRS	SCZ spectrum disorders patients	PC and GPC (basal ganglia) ↓; PC, PE(frontal and temporal cortex) ↓	Brain Energy Metabolism, Myelination	[104]
High-throughput lipidomics	SCZ patients (Red blood cell)	PC ↑ (gray matter); PC ↓, ceramide ↑ (white matter); FFA ↑ (both gray and white matter)	Oxidative stress, mitochondrial dysfunction, neuroinflammation	[103]
Metabolomics	SCZ patients (Serum, Urine)	Fatty acids ↑, ketone bodies ↑ (e.g., β-HB, pyruvate)	Abnormal glucose and fatty acid metabolism	[108]
Colorimetry	SCZ patients	β-HB ↑	Brain Energy Metabolism	[109]
31P-MRS	Untreated SCZ patients	PME/PDE ratio (basal ganglia) ↑	Neurodevelopmental abnormalities	[113]
31P-MRS	First-episode drug-naive SCZ patients	GPC ↑ (ACC)	Enhanced membrane phospholipid degradation	[111]
31P-MRS	Drug-naive SCZ patients	PME, PDE ↓	Membrane phospholipids dysfunction, myelin sheath dysfunction	[112]
31P-MRS	Drug-naive SCZ patients	PDE ↓	Impaired Membrane phospholipids, impaired energy metabolism	[114]
31P-MRS	First-episode drug-naive SCZ patients	(Right prefrontal) PDE ↑, (left prefrontal) PME ↑	Glutamate ↑, impaired membrane phospholipid metabolism	[116]
LC-MS/MS	Nrg1 heterozygous mice	2-AG ↑ (hippocampus)	Impairing spatial learning	[118]
Proteomics	Stable-phase SCZ patients	TG ↑, TC ↑, LDL-C ↑	High TG and LDL-C, cognitive deterioration	[121]
Lipidomics	Finnish twins with SCZ	LPC ↓	Insulin resistance	[125]
LC-MS lipidomics	First-episode SCZ patients	n3 PUFAs ↓	Abnormal lipid metabolism	[127]
Lipidomics	SCZ patients (American)	PE ↓, PUFA ↓	Impaired nerve conduction	[122]
Mass spectrometry lipidomics	SCZ patients (Plasma, platelets)	Plasma choline, ethanolamines ↓	Lipid transport/remodeling disorders	[123]
Metabolomics	SCZ patients (Finland)	Saturated triglycerides ↑	Insulin resistance, lipid metabolism disorders	[128]
Lipidomics of erythrocyte membrane	Stable-phase SCZ patients	SM ↓	Impaired Dopamine signaling, cognitive Dysfunction	[129]
LC-MS targeted lipidomics	SCZ patients	FFAs ↑	Lipid mobilization, oxidative stress	[130]
Lipid profile analysis	First-episode drug-naive SCZ patients	TC, TG, LDL ↑ (male patients with impaired glucose tolerance)	lipid metabolism	[131]
Erythrocyte membrane lipidomics	SCZ patients	PC ↓, PE ↓	Oxidative stress, lipid peroxidation, decreased membrane fluidity	[132]
Multi-omics integration	SCZ patients	Anti-inflammatory lipids ↓ (such as oleic acid, linoleic acid, arachidonic acid)	Intestinal flora dysregulation, inflammatory responses	[133]
Lipidomics	First-episode SCZ patients (Serum)	16 kinds of PCs, SM-C ↓	Mitochondrial fatty acid oxidation disorder, inflammatory response	[134]

Free fatty acids (FFAs), polyunsaturated fatty acids (PUfAs), N-acyl phosphatidylserine (NAPS), n-acyl serine (NAS), N-acyl phosphatidylethanolamine (NAPEs), n-acylethanolamine (NAEs), N-acetyl aspartic glutamate (NAAG), phosphatidylcholine (PC), glycerol phosphocholine (GPC), phospholipid monoester (PME), phospholipid diester (PDE), phosphatidylethanolamine (PE), 2-arachidonyl glycerol (2-AG), sphingomyelin (SM), β-hydroxybutyric acid (β-HB), anterior cingulate gyrus (ACC), lysophosphatidylcholine (LPC).

**Table 3:** Glutamate and related amino acid metabolism in schizophrenia.

Method	Subjects	Abnormal Indicators	Mechanisms/ Effects	Refs.
Western blot	Prenatal MAM model rats (P7-P45)	NR3A protein ↓, (early adolescence); NR2B protein ↓ (adolescence)	NMDA dysfunction, impaired spatial learning	[152]
In situ hybridization + receptor autoradiography	SCZ postmortem hippocampus (CA3, DG)	NMDAR1 mRNA ↓ (left CA3/DG)	Glutamatergic signaling impairment	[153]
In situ hybridization + receptor autoradiography	SCZ patients (prefrontal cortex) *n* = 15	NR1, NR2A, NR2C mRNA ↓	Imbalance in NMDA receptor subunit ratios	[154]
SPET	Untreated SCZ patients, brain (*n* = 5)	Left hippocampal NMDA receptor binding ↓	NMDA receptor functional defect	[155]
PET-MR imaging	First-episode psychotic patients, hippocampus (*n* = 21)	NMDA receptor availability ↓	Reduced NMDA receptor	[156]
Western blot,	MAM neurodevelopmental rat model	NR2B protein ↓	Impaired NMDAR function	[158]
Microdialysis	PCP/MK-801 subchronic treatment model	d-Ser, Glu, DA, Ach ↓	NMDAR antagonism, cognitive impairment	[159]
Targeted proteomics	SCZ patients, brain (*n* = 22)	GRIA3, GRIA4, ATP1A3 ↓	Altered synaptic protein	[160]
ELISA	Egyptian SCZ patients (*n* = 100)	d-Ser ↓, DAAO ↑	Abnormal d-Ser, impaired NMDAR function	[163]
Genotyping, MRI	SCZ patients, brain (*n* = 52)	d-Ser ↓	Weakened NMDAR function	[172]
Vitro experiments	SCZ patients	Three pLG72 variants (R30, R30K, K62E) d-Ser levels ↓	D-serine overdegradation, NMDAR function impairment	[173]
Targeted exome resequencing, metabolomics	SCZ patients, brain (*n* = 474)	Aspartic acid ↑, glutamic acid ↑, 5-oxoproline ↑	GCS functional defect, NMDAR dysfunction	[179]
Animal studies	SCZ rat model (CSF)	Glycine levels ↑ (dose-dependent increase)	Enhances NMDAR function	[180]
High-performance liquid chromatography	SCZ patients, brain (*n* = 15)	NAA and NAAG ↓ (temporal cortex)	Impaired neuronal function	[189]
qRT-PCR, fMRI	Postmortem SCZ patients (prefrontal cortex), Ddo-/- mice	D-Asp ↓; DDO mRNA ↑	Reduced D-Asp, impaired NMDA function	[192]
qPCR detection	SCZ peripheral blood leukocytes (*n* = 96)	mRNA: SLC3A2 and SLC7A11 ↓	Oxidative stress, glutamatergic dysfunction	[195]
In situ hybridization, Western blot	Elderly SCZ patients (Frontal and cingulate cortex)	protein: EAAT1 (frontal cortex) ↓, EAAT3 (cingulate cortex) ↑	Reduced glutamate reuptake capacity,	[197]
Genotyping	Stable-phase SCZ patients (*n* = 192)	EAAT1 (T allele) and EAAT2 (G allele) ↓	Impaired energy metabolism	[198]
Genotyping, MRI structural imaging	Chronic SCZ patients (*n* = 50)	EAAT2 (G allele carriers) ↓	Reduced glutamate clearance/gray matter volume	[199]
Genotyping	Stable-phase SCZ patients (*n* = 211)	EAAT2 (G allele carriers) ↓	Reduced glutamate clearance/gray matter volume	[200]
3T 1H-MRS	SCZ patients, DLPFC and ACC (*n* = 25)	Decreased NAA/Cr and Glx/Cr ratios in DLPFC	Glutamate pathway dysfunction, oxidative stress	[204]
7T 1H-MRS	SCZ patients, brain (*n* = 24)	Increased age associated with Glu in ACC ↓	Decreased glutamate	[208]
1H-MRS	SCZ patients (*n* = 188)	Glu ↓ (prefrontal ACC and ACC)	Abnormal glutamate-glutamine cycle	[209]
9.4T 1H-MRS	PCP model rats, PFC	Glu ↑, Gln ↓, Gln/Glu ↑, GABA ↓	Abnormal glutamate-glutamine cycle	[210]
7T 1H-MRS	Ketamine-induced social isolation rats, PFC	GABA ↓, GABA/Gln ↓	NMDA antagonism, GABA pathway defect	[211]
3T 1H-MR	14 pairs of SCZ discordant twins	Medial prefrontal cortex Glu ↓	Decreased glutamate	[214]
1.5T 1H-MRS	Brain tissue adolescents at high risk for SCZ (*n* = 40)	NAA ↓ (caudate), Glu + Gln ↓ (male caudate), NAA ↑ (frontal white matter)	Reduced caudate neuronal function,	[215]
3T 1H-MRS	SCZ patients, GM and WM (*n* = 104)	Glx ↑ (GM and WM), NAAc ↑ (GM, elderly), NAAc ↓ (WM, elderly)	Enhanced glutamate metabolism	[216]
HPLC detection	Male SCZ patients, CSF (on olanzapine, *n* = 16)	KYN ↑, KYNA ↑	Possibly due to TDO ↑ or KMO ↓	[225]
RT-PCR	Postmortem SCZ patients, anterior cingulate cortex (*n* = 12)	TDO2 ↑, KYN ↑	Increased conversion of tryptophan to KYN	[227]
Targeted metabolomics, genotyping	SCZ patients, CSF (*n* = 17)	KYNA ↑	KMO polymorphism	[229]
HPLC detection	First-episode/discontinued SCZ patients, plasma (*n* = 53)	KYNA, 3-HK ↑, KYNA/3-HK ↓	Pro-inflammatory state activates IDO/KMO	[231]
PCR	SCZ patients, saliva (pre/post-stress, *n* = 64)	KYNA ↑ 20 min post-stress	Abnormal tryptophan metabolism	[232]
Cell experiments	SCZ patients, peripheral skin fibroblasts (*n* = 11)	Baseline 3-HK ↑; further 3-HK ↑ after cytokine stimulation	Inflammatory response, Abnormal tryptophan metabolism	[233]
Liquid chromatography-mass spectrometry	Stable-phase SCZ patients, CSF (*n* = 22)	KYNA and xanthine ↑; QUIN/KYNA ratio ↓	Tryptophan metabolism directed toward KYNA pathway	[234]
Metabolomics	SCZ patients, Plasma (*n* = 139)	3-OHKY, XANU ↓; XAN ↑	Dysregulated tryptophan metabolism/purine metabolism	[235]
Targeted metabolomics	First-episode, untreated SCZ patients, plasma (*n* = 25)	NAS ↑; correlation between 5-HIAA and tryptophan ↓	Impaired 5-HT metabolic pathways	[236]
ELISA	Chronic SCZ patients, CSF (on olanzapine, *n* = 23)	IL-6 ↑, KYNA ↑, KYN ↑	Abnormal tryptophan-kynurenine pathway	[240]
In situ hybridization + autoradiography	Postmortem SCZ patients, brain (*n* = 10)	mRNA-positive neuron density for GAT-1 in prefrontal cortex ↓	Impaired synaptic GABA clearance	[243]
In situ hybridization + autoradiography	Postmortem SCZ patients, brain (*n* = 10)	GAD67 mRNA-positive neuron density in prefrontal cortex ↓	weakened GABAergic inhibition	[244]
1H-MRS	Chronic SCZ patients, brain (*n* = 17)	GABA/Cr ratio in prefrontal cortex ↓	Impaired NMDA receptor function	[245]
PET	Ultra-high-risk individuals	GABA-A/BZ receptor binding ↓ (right caudate nucleus)	excitation-inhibition imbalance	[246]
Case–control studies	SCZ patients	GSH ↓, MDA ↑, NO ↑, SOD ↓	Enhanced oxidative stress	[250]
14.1T MRS	GCLM-KO SCZ model mice (simulating GSH deficiency)	GSH ↓, Glu ↑, Gln ↑, Gln/Glu ↑, NAA ↑	Glutamate accumulation, mitochondrial dysfunction	[251]
Neurochemical analysis	Transgenic mice (mimicking schizophrenia)	GSH ↓, GAD67 ↓, Reelin ↓	Oxidative stress, GABAergic neuronal dysfunction	[254]
MRS	GCLM-KO SCZ model mice	GSH ↓, Glu ↑, Gln ↑, NAA ↑	Oxidative stress, glutamate homeostasis	[252]
MRS	SCZ patients, brain	GSH ↓ (prefrontal cortex)	Oxidative stress, NMDA receptor dysfunction	[253]
ELISA	SCZ model mice	GSH ↓	Oxidative stress, inflammation, monoamine imbalance	[255]

Single-photon emission tomography (SPET), d-serine (d-Ser), glutamate (Glu), dopamine (DA), acetylcholine (Ach), N-acetyl aspartate (NAA), N-acetyl aspartyl glutamate (NAAG), D-aspartic acid (D-Asp), D-aspartate oxidase (DDO), dorsolateral prefrontal cortex (DLPFC), anterior cingulate cortex (ACC), glutamine (Gln), prefrontal cortex (PFC), gray matter (GM), white matter (WM), 3-hydroxykynurenic acid (3-OHKY), xanthine (XANU), xanthine (XAN), N-acetyl serotonin (NAS), 5-hydroxyindoleacetic acid (5-HIAA), GABA Transporter 1 (GAT-1), glutathione (GSH), Glutamate Decarboxylase 67 (GAD67), cerebrospinal fluid (CSF).

**Table 4:** Carbon metabolism in schizophrenia.

Method	Subjects	Abnormal Indicators	Mechanisms/ Effects	Refs.
Mendelian randomization analysis	GWAS data	Hcy ↑ (genetic prediction)	NMDA receptor activation, neurotoxicity	[273]
Case–control study	Treat-naive first-episode psychotic patients, India, Plasma (*n* = 31)	Folate, vitamin B12, DHA ↓; Hcy, cortisol ↑	One-carbon metabolism disorder, oxidative stress, abnormal DNA methylation	[275]
qPCR	Treat-naive first-episode SCZ patients, Plasma (*n* = 52)	mRNA: serum Hcy genes ↑	One-carbon metabolism disorder, oxidative stress, abnormal DNA methylation	[277]
Intervention study (risperidone treatment)	Chinese first-episode, treatment-naive SCZ patients, Plasma (*n* = 56)	Hcy ↓(post-treatment)	Hcy metabolism	[278]
Case–control study	Polish first-episode SCZ patients, Plasma (*n* = 56)	Hcy ↑; folate, B12, HDL ↓	Metabolic dysfunction, neurotoxicity	[279]
Cross-sectional study	Han Chinese SCZ patients, Plasma (18–59 years, *n* = 103)	Hcy ↑	NMDAR dysfunction, oxidative stress, mitochondrial damage	[280]
Genotyping	Han Chinese SCZ patients, Serum (*n* = 715)	Hcy ↑, MTHFR C677T genotype	NMDA receptor antagonism	[281]
Whole-genome DNA methylation array	Japanese male chronic SCZ patients, Plasma (*n* = 42)	Hcy ↑	Abnormal whole-genome DNA methylation	[282]
Mendelian randomization analysis	Japanese SCZ patients, Serum (*n* = 365)	Pyruvate ↓	Impaired one-carbon metabolism	[276]

Homocysteine (Hcy).

**Table 5:** Effects of electroconvulsive therapy on the neuro–immune–endocrine system in schizophrenia.

Subjects	ECT Parameters	Three System Indicators	Therapeutic Outcomes	Mechanisms of ECT	Refs.
SCZ patients, mPFC (*n* = 14)	8–12 sessions, 3 times/week, bilateral temporal electrodes, 504 mC, 0.9 A, 10–70 Hz, 0.5 ms pulse width, 8 s duration	GABA+/Cr ↑ (ECT group only)	PANSS improvement	Enhanced GABA-mediated neuroinhibition	[295]
Refractory SCZ patients, peripheral blood (*n* = 8)	6 sessions, 2 weeks, bilateral temporal electrodes, 504 mC, 0.9 A	Gene expression ↑ (neurotrophic factor, MAPK, LTP pathway)	PANSS improvement rate consistently > 50%	Neuroplasticity-related gene expression	[296]
SCZ patients (bilateral hippocampus/parahippocampal gyrus, right insula, right temporal pole) (*n* = 42)	8–12 sessions, 3 times/week, bilateral temporal electrodes, 504 mC, 0.9 A	GMV ↑	Hippocampal GMV ↑ positively correlated with positive symptom improvement	Induces neuroplasticity	[297]
SCZ patients, right thalamus-right putamen, thalamus-sensory cortex (*n* = 21)	Same as first, 504 mC, 0.9 A, 10–70 Hz, 1.0 ms pulse width, 8 s duration	Right thalamus-right putamen FC ↑; thalamus-sensory cortex connectivity ↓	PANSS total score ↓	Restoration of cortical-striatal-thalamic-cortical circuit balance	[298]
SCZ patients, DMN (*n* = 21)	4 weeks, average 11.5 treatments, 504 mC, 0.9 A, 10–70 Hz	DMN (dMPFC, vMPFC, Pcu) global FC density ↑	No significant difference in PANSS scores ↓ between ECT and medication-only groups	DMN hyperconnectivity; neuroplasticity mechanism	[299]
SCZ patients, DMN (*n* = 21)	4 weeks, 8–12 treatments, 504 mC, 0.9 A, 10–70 Hz	FC↑ in the AG-MTG region (left AG-right MTG significantly)	Reduced PANSS negative symptom scores correlated with AG-MTG FC ↑	DMN hyperconnectivity, negative symptoms	[300]
SCZ patients, bilateral hippocampus (*n* = 21)	12 sessions, 3 times weekly, 504 mC, 0.9 A, 10–70 Hz	Hippocampal volume, cognitive network FC ↑	Symptom improvement	Neuroplasticity, symptom remission	[301]
SCZ patients, cerebellum-cerebral circuit (*n* = 21)	4 weeks, 8–12 treatments, 504 mC, 0.9 A, 10–70 Hz	FC ↑ (especially left cerebellum-temporal lobe)	Reduced PANSS psychotic symptom subscale correlated with FC changes	Restored cerebellum-cerebral circuit function	[302]
SCZ patients, DMN, temporal lobe/language network (*n* = 13)	Average 9.1 treatments	DMN, language network FC ↑; cortical-striatal network FC ↓	PANSS total score ↓ 31%, negative symptom score ↓ 18%	FC patterns shifted toward healthy state	[303]
SCZ patients, DMN, striatal network, executive/salience network (*n* = 8)	Average 12.4 treatments, right unilateral ECT	DMN-mPFC FC ↑; Striatal low-frequency oscillations ↓	PANSS total score ↓ 38%	Dopamine signaling modulation (D2/D3 receptors)	[305]
Gunn rat model (*n* = 8)	Daily for 6 days: 100 V, 60 Hz, 50 mA, 1.5 s	CD11b ↓ (glial cells), GFAP ↓ (astrocytes), PPI ↑	Improved schizophrenia-like behavior	Suppressed glial activation, reduced neuroinflammation	[308]
MK-801 rat model, prefrontal cortex (*n* = 4)	Daily for 10 days, 55 mA, 100 Hz, 0.5 ms	Egr1 ↓, Mmp9 ↑, S100a6 ↑; IL-17, NF-κB, TNF pathway activation ↓	Improved PPI deficits (restored sensory gating)	Anti-inflammatory, insulin signaling, and mitochondrial autophagy pathway regulation	[310]
SCZ patients (*n* = 31)	Bilateral temporal electrodes, short-pulse current, 6–12 treatments	Total leukocytes ↑, neutrophils ↑, lymphocytes ↓, hemoglobin ↑	Therapeutic efficacy not evaluated	Stress response activates HPA axis, catecholamine release	[311]
SCZ patients (*n* = 6)	Single treatment, bilateral temporal electrodes, average energy 34 J	NF-κB, iNOS, PGE2 ↓ (responders)	Reduced oxidative stress in clinical (responders), increased (non-responders)	Anti-inflammatory, antioxidant mechanisms, NF-κB pathway downregulation	[312]
Refractory SCZ patients (*n* = 20)	3 sessions weekly, 9 total treatments, bilateral frontal-temporal electrodes, 504 mC	IL-4 ↑, TGF-β ↑; NF-κB activation, no change in MPO	Improvement in BPRS scores, TGF-β ↑ negatively correlated with symptom improvement	Upset anti-inflammatory factors, immune imbalance regulation	[313]
Refractory SCZ patients (*n* = 31)	Every other day, average 13 sessions, bilateral temporal electrodes, 0.9 A, 70 Hz	TNF-α ↓, no significant changes in VEGF or BDNF	PANSS total score ↓; reduced TNF-α correlated with treatment efficacy	Anti-inflammatory effect	[314]
SCZ patients (Japan, *n* = 13)	6-month titration method for ECT intensity up to 3 sessions weekly, total 3–15 sessions	Serum MMP-9 levels ↓	PANSS/BPRS scores ↓ (84.6% efficacy)	Inflammatory factors	[316]
SCZS patients (China, *n* = 28)	6 sessions, 2–3 times weekly	IL-18 mRNA ↓, KYN/TRP ↓ (significant reduction only in low-inflammation group)	Low-inflammation group showed greater PANSS score reduction	Inhibiting inflammation, tryptophan-kynurenine pathway	[9]
Refractory SCZ patients (China, *n* = 40)	Average 6.76 ECT sessions, 3 times weekly, bilateral frontal-temporal electrodes	Serum VEGF levels ↑ (significantly elevated post-ECT)	PANSS score ↓, 60% of patients responded	Neurovascular repair, blood–brain barrier regulation	[317]
Male SCZ patients (Italy, *n* = 11)	8 bilateral ECT sessions, 3 times weekly	Serum GH ↓ (significant decrease 15–30 min post-ECT)	Improvement in PANSS total score, positive and negative symptoms	GH decrease	[322]

Whole-brain gray matter volume (GMV), default mode network (DMN), anterior cingulate—middle temporal gyrus connectivity (AG-MTG), functional connectivity (FC), medial prefrontal cortex (mPFC), nuclear factor kappa-B (NF-κB), inducible nitric oxide synthase (iNOS), prostaglandin E-2 (PGE2), matrix metalloproteinase-9 (MMP-9), interleukin-17 (IL-17), tumor necrosis factor (TNF), myeloperoxidase (MPO), kynurenine (KYNA), vascular endothelial growth factor (VEGF), growth hormone (GH).

## Data Availability

No new data were created or analyzed in this study. Data sharing is not applicable to this article.
